# “Proteotranscriptomic analysis of advanced colorectal cancer patient derived organoids for drug sensitivity prediction”

**DOI:** 10.1186/s13046-022-02591-z

**Published:** 2023-01-06

**Authors:** Federica Papaccio, Blanca García-Mico, Francisco Gimeno-Valiente, Manuel Cabeza-Segura, Valentina Gambardella, María Fernanda Gutiérrez-Bravo, Clara Alfaro-Cervelló, Carolina Martinez-Ciarpaglini, Pilar Rentero-Garrido, Sheila Zúñiga-Trejos, Juan Antonio Carbonell-Asins, Tania Fleitas, Susana Roselló, Marisol Huerta, Manuel M. Sánchez del Pino, Luís Sabater, Desamparados Roda, Noelia Tarazona, Andrés Cervantes, Josefa Castillo

**Affiliations:** 1 Department of Medicine, Surgery and Dentistry “Scuola Medica Salernitana”, University of Salerno, Via S. Allende, 84081 Baronissi, Italy; 2 Department of Medical Oncology, Hospital Clínico Universitario de Valencia, INCLIVA Biomedical Research Institute, University of Valencia, Avda. Blasco Ibañez 17, 46010 Valencia, Spain; 3 University College London Cancer Institute, Cancer Evolution and Genome Instability Laboratory, London, UK; 4 Centro de Investigación Biomédica en Red (CIBERONC), Instituto de Salud Carlos III, 28029 Madrid, Spain; 5 Health Sciences Faculty, Universidad Internacional SEK (UISEK), Quito, 170120 Ecuador; 6 Department of Pathology, Hospital Clínico Universitario, INCLIVA Biomedical Research Institute, University of Valencia, Avda. Blasco Ibañez 17, 46010 Valencia, Spain; 7 Precision Medicine Unit, INCLIVA Biomedical Research Institute, University of Valencia, Avda. Blasco Ibañez 17, 46010 Valencia, Spain; 8 Bioinformatic and Biostatistic Unit, INCLIVA Biomedical Research Institute, University of Valencia, Avda. Blasco Ibañez 17, 46010 Valencia, Spain; 9 University Institute of Biotechnology and Biomedicine (BIOTECMED), University of Valencia, 46100 Burjassot, Spain; 10 Department of Biochemistry and Molecular Biology, Universitat de València, 46100 Burjassot, Spain; 11 Liver, Biliary and Pancreatic Unit, Department of Surgery, Hospital Clínico Universitario of Valencia, University of Valencia, INCLIVA Biomedical Research Institute, University of Valencia, Avda. Blasco Ibañez 17, 46010 Valencia, Spain

**Keywords:** Colorectal cancer, Organoids, Quantitative proteomics, Precision medicine, Drug resistance, Transcriptomics, Proteotranscriptomics integrative functional network analysis

## Abstract

**Background:**

Patient-derived organoids (PDOs) from advanced colorectal cancer (CRC) patients could be a key platform to predict drug response and discover new biomarkers. We aimed to integrate PDO drug response with multi-omics characterization beyond genomics.

**Methods:**

We generated 29 PDO lines from 22 advanced CRC patients and provided a morphologic, genomic, and transcriptomic characterization. We performed drug sensitivity assays with a panel of both standard and non-standard agents in five long-term cultures, and integrated drug response with a baseline proteomic and transcriptomic characterization by SWATH-MS and RNA-seq analysis, respectively.

**Results:**

PDOs were successfully generated from heavily pre-treated patients, including a paired model of advanced MSI high CRC deriving from pre- and post-chemotherapy liver metastasis. Our PDOs faithfully reproduced genomic and phenotypic features of original tissue. Drug panel testing identified differential response among PDOs, particularly to oxaliplatin and palbociclib. Proteotranscriptomic analyses revealed that oxaliplatin non-responder PDOs present enrichment of the t-RNA aminoacylation process and showed a shift towards oxidative phosphorylation pathway dependence, while an exceptional response to palbociclib was detected in a PDO with activation of MYC and enrichment of chaperonin T-complex protein Ring Complex (TRiC), involved in proteome integrity. Proteotranscriptomic data fusion confirmed these results within a highly integrated network of functional processes involved in differential response to drugs.

**Conclusions:**

Our strategy of integrating PDOs drug sensitivity with SWATH-mass spectrometry and RNA-seq allowed us to identify different baseline proteins and gene expression profiles with the potential to predict treatment response/resistance and to help in the development of effective and personalized cancer therapeutics.

**Supplementary Information:**

The online version contains supplementary material available at 10.1186/s13046-022-02591-z.

## Background

Colorectal cancer (CRC) is the third most common and second deadliest cancer [1]. Even when diagnosed at early stage, 30–50% of patients will experience relapse and die of the disease [2]. Whether relapsed or diagnosed as stage IV disease, survival has significantly improved over the last ten years, going from 6 to more than 30 months median overall survival [3]. However, despite improvements in cancer therapy, resistance to chemotherapeutic and novel targeted therapies limits treatment success.

In light of the increasing notion of CRC molecular complexity, the role of useful preclinical models for predictive biomarkers and new drug discovery is gaining importance. Furthermore, there is an urgent need to go beyond genomics, as its utility to guide therapy has been less successful than anticipated [4]. Indeed, the presence of a molecular alteration at the genomic level does not automatically predict treatment response, as multiple factors can influence response in each individual patient to a greater or lesser extent [5].

Functional testing using human-derived cancer models offers a valuable complementary approach to help in personalizing treatments. Patient-derived organoids (PDOs) are employed to fill the gap left by traditional preclinical models. They recapitulate the biology of original tumor tissue, including therapy response [6], and can be rapidly generated for many cancer types [7–10] with a lower cost than animal models. A great number of PDO models have been generated for CRC [6, 11–13]. However, the evidence obtained until now with these models comes mainly from cultures deriving from primary tumors, while data obtained from metastatic samples are less consistent.

Understanding the phenotypic alterations that play a role in differential response to drugs will be pivotal in identifying predictive biomarkers and developing efficacious therapies for advanced CRC. The bulk of research to date has focused on the value of organoids as a predictive tool [6, 12, 14]; some have characterized the transcriptomic profile in relation to drug sensitivity [13, 15] in an effort to identify predictive biomarkers, but only a limited number of studies have used quantitative proteomic analysis to characterize PDOs [16, 17]. Extensive literature indicates that transcriptomic and proteomic expression profiles lack correlation due to significant post-transcriptional regulation, which could explain why RNA signatures are rarely useful as drug response predictors in the clinic [18].

In this study we generated PDO models from advanced CRC patients including heavily pre-treated patients and a model of microsatellite unstable- (MSI) high CRC, for which pre- and post-chemotherapy liver disease PDOs were established and characterized to ensure biological fidelity with the original tumors. Our main objective was to integrate functional drug assays conducted on PDOs with proteotranscriptomic study to explore the mechanisms underlying drug response. To this end, we used SWATH-MS (sequential window acquisition of all theoretical mass spectrometry), a highly accurate proteomic quantification technique and a bulk RNA-sequencing analysis and performed an integrative functional network analysis of both omics. This strategy could represent a useful tool to discover new therapeutic targets in advanced CRC and to guide personalized therapies.

## Methods

### Tissue processing and organoid culture

Locally advanced or metastatic colorectal samples were collected after patients had signed written informed consent at Hospital Clínico Universitario de Valencia (HCUV). The HCUV Ethics Committee (EC) approved the study (2018/063, 2021/083). Samples were collected at surgery or with image-guided tissue biopsy. Both naïve and pre-treated patients were included.

Fresh tissues were collected in PBS with antibiotics and quickly processed as follows. After serial washings and antibiotic incubation, tissue samples were minced in small fragments. Some of them were stored for DNA and RNA extraction depending on the total amount of tissue. All the remaining fragments were mechanically and enzymatically digested with a collagenase-based solution (Sigma-Aldrich, cat. No. 269395). Subsequently, the resulting digestion solution was filtered, single cells were counted, resuspended in basal media with 50% reduced-growth factor basement membrane matrix (BME Type 2, R&D, cat. No. 3533–010-002), plated in prewarmed culture plates and stored at 37 °C. After the formation of domes, complete medium was added.

Complete medium composition was as follows: 1 × N2 (cat. No. 17502048), 1 × B27 (cat. No. C21-H), 1 mM N-acetyl L-cysteine (cat. No. A9165), 10 mM nicotinamide (cat. No. N0636), 50 ng/mL hEGF (cat. No. BMS320), 100 ng/mL hNoggin (cat. No. 6057-NG-025), 0.5 mM A-83–01 (cat. No. SML0788), 10 mM SB202190 (cat. No. S7067), 10 nM gastrin (cat. No. G9145), 500 ng/mL hRSPO1 (cat. No. 4645-RS), 10 ng/mL hFGF10 (cat. No. 100–26), 10 nM PGE2 (cat. No. P6532), 10 mM Y-27632 (cat. No. Y0503).

Medium was changed three times a week. Organoid’s formation was observed in a fraction of cultures (see Results section). After reaching appropriate volume organoids were trypsinized with TrypLE™ Express (Life Technologies, cat. No. 12605–010) to expand them. Aliquots were stored in liquid nitrogen to constitute a Biobank.

All samples were named with a codified nomenclature indicating the site of biopsy (metastasis or primary) and with a serial progressive number: metastatic colon tumor organoid (mCTO), metastatic colon tumor tissue (mCTT), rectal tumor organoid (RTO) rectal tumor tissue (RTT) colon normal organoid (CNO) and colon normal tissue (CNT). Collectively, all models are referred as patient-derived organoids (PDOs).

### Morphologic characterization

PDOs domes were collected in 4% neutral buffered formalin and paraffin embedded (FFPE). 4 mm slides were cut, dewaxed and hematoxylin and eosin staining (Dako, cat. No. CS700 and CS701) was performed. For IHC sodium citrate antigen retrieval was performed (Target Retrieval Solution, Citrate pH 6, S236984-2 and pH 9, S236784-2), followed by peroxidase blocking (DakoREAL™, Dako, cat. No. S2023) and by incubation with the following primary antibodies in EnVision FLEX antibody diluent (Dako, cat. No. K8006): Ki67 (Dako, cat. No. IR626, ready to use), CDX2 (Dako, cat. No. IR080, ready to use), CK20 (Dako, cat. No. IR777, ready to use). In some PDOs the following additional stainings have been perfomed: ERBB2 (Roche, cat. No. 790–2991, 4B5 clone), PTEN (Dako, cat. No. M3627, 1:50). Peroxidase-conjugated secondary antibodies were used (EnVision Flex/HRP, Dako, cat.no. GV82311-2). The slides were also counterstained with hematoxylin.

Mismatch repair proteins MLH1 (Dako, cat. No. 7R079, ready to use), PMS2 (Dako, cat. No. 7R087, ready to use), MSH2 (Dako, cat. No. IR086, ready to use), MSH6 (Dako, cat. No. IR085, ready to use), PDOs’ staining was performed when clinical data indicated that the patient had a loss of expression of these proteins.

The same procedures were performed also for FFPE tissue sections. All slides from both PDOs and tissues were independently reviewed by dedicated pathologists.

### Genomic characterization

Fresh tissues and PDOs DNA was extracted with the QIAamp DNA Micro Kit (Qiagen, cat. No. 56304) to perform both targeted next-generation sequencing (NGS) and copy number variation analysis with CytoScan HD. NGS was performed using OncoSpot v1, an in-house customized 87-gene panel [19]. A minimum of 80 ng of DNA was used for library preparation, using KAPA HyperPlus Library Preparation (Roche Diagnostics). Libraries were paired-end sequenced in an Illumina MiSeq platform with a MiSeq Reagent Kit v2 (300-cycles) kit.

Read quality control was performed with FASTQC v0.11.8 (available online at: http://www.bioinformatics.babraham.ac.uk/projects/fastqc/). Sequencing adapters and low-quality reads were filtered out with fastp v0.11.8 [20]. Filtered reads were aligned against the human reference genome GRCh38 with BWA-mem v0.7.17 (Li, H. (2013). Aligning sequence reads, clone sequences and assembly contigs with BWA-MEM. arXiv: Genomics). PicardTools v2.18.6 (http://broadinstitute.github.io/picard) was applied to eliminate duplicated reads. A combination of LoFreq v2.1.3.1 [21] and Mutect2 v 4.0.5.0 [22] was used to call variants for individual patients. Variants were merged with vcftools [23] and normalized to avoid multi-allelic positions with BCFtools [24]. Then, variants were functionally annotated using Variant Effect Predictor v96 [25]. Additional information was added from the Cancer Hotspot database [26] and a proprietary cancer mutation database containing information from different resources such as PCT MDAnderson database (https://pct.mdanderson.org), commercial cancer panels (Oncomine® and Sequenom), relevant literature and our own expertise. Variants with a depth of coverage greater than 100 × and an allelic fraction of 5% or higher were reported. Pathological variants were selected if present in our proprietary database or located in a cancer hotspot with a population frequency below 1%. Discordant results and low VAFs were manually curated with IGV, v. 2.9.4. Pathogenic variants were summarized using the ComplexHeatmap (v2.4.3) R package [27]. Data available after acceptance.

For copy-number analysis, hybridization-based genomic profiling was performed using CytoScan HD Array (Affymetrix, CA, USA) according to manufacturer’s protocol. CytoScan HD CEL files were processed through Chromosome Analysis Suite (ChAS) software version 4.1 with a single sample algorithm. All samples were manually reviewed, and unbalanced samples were reprocessed with normal diploid algorithm. Obtained Chyp files were analyzed with ChAS and IGV software v. 3.0. Copy number and loss of heterozygosity (LOH) data were retrieved for further analysis.

### RNA-sequencing analysis (RNA-seq)

Total RNA was extracted from fresh tissues and PDOs with RNeasy Micro Kit (cat. no. 74004) and integrity verified with TapeStation RNA Analysis ScreenTape (Agilent Technologies). Sequencing libraries were prepared with the NEBNext® Ultra (TM) II Directional RNA Library Prep Kit for Illumina® Module (New England BioLabs) and NEBNext Poly(A) mRNA Magnetic Isolation Module for mRNA enrichment following the manufacturer's instructions. Libraries were paired end sequenced in an Illumina NextSeq 550 platform with a NextSeq 500/550 High Output 300 cycles kit.

Sequence quality control was performed as in gene panels. Filtered reads were mapped against the human reference GRCh38 genome using STAR v2.7.3a [28]. Isoform quantification was performed with RSEM v1.3.3 [29] and further processed with Tximport v1.16.1 [30] to summarize counts per gene. Differential expression analysis was conducted with the DESeq2 v1.28.1 package [31] using an adjusted *p*-value cutoff of 0.05.

Gene-set enrichment analysis (GSEA) analysis [18] was run against the hallmark gene sets from the Human Molecular Signatures Database (MSigDB). The significance threshold was set at *P*-value below 0.05 and nominal false discovery rate (FDR) below 0.05.

To determine the agreement between gene expression in tumor tissues and their derived organoids, first stromal genes described in Isella et al. [32] and non-protein coding genes were removed. To minimize the effect of the artificial environment in which organoids are grown, differentially expressed genes between tumor tissues and organoids were identified with DESeq2 and filtered out. The remaining genes were used to calculate Pearson correlation coefficients between organoids and tissues and were plotted in a heatmap along with an unsupervised hierarchical clustering using the heatmap v1.0.12 R package (https://CRAN.R-project.org/package=pheatmap).

## Droplet-digital PCR

DNA from PDOs was digested with the restriction enzymes Mse I (TakaRa, cat. No. 1247A) and Hae III (TakaRa, ca. no. 1051A). PCR droplets were generated using the QX200 droplet generator (Bio-Rad), and the PCR reaction was run in a C1000 Touch thermal cycler (Bio-Rad) according to the manufacturer’s protocol. Results were analyzed with the QuantaSoft software (Bio-Rad). The following probes have been purchased from Bio-Rad: *ERBB2* (cat. No. dHsaCP1000116), *TP53* (cat. No. dHsaCP1000586), *AURKA* (cat. No. dHsaCNS193384431), *CDKN2A* (cat. No. dHsaCP1000581), *FGFR1* (cat. No. dHsaCP2500319), *MET* (cat. No. dHsaCP2500321), *SMAD4* (cat. No. dHsaCP2500468). *AGO2* was used as a reference assay (cat. No. dHsaCP2500349). DNA from fresh normal mucosa tissue was used as diploid control sample.

### Proteomics analysis by LC–MS/MS-SWATH

#### Sample preparation

PDOs pellets were lysed in UTC buffer (7 M urea, 2 M thiourea and 4% CHAPS) and the protein concentration was measured with the RC DC Protein Assay (Bio-Rad). A pooled sample containing 5 µg of protein from each sample was used to prepare the peptide library. For the SWATH analysis, 20 µg of protein from each sample were used. Samples were denatured at 95 °C for 5 min in sample buffer and subjected to sodium dodecyl sulfate–polyacrylamide gel electrophoresis (SDS-PAGE). Pooled samples were resolved in gel and lanes were cut into five pieces. Gels containing individual samples were not resolved and whole samples were sliced into a single piece. Protein digestion and subsequent analysis were performed as published elsewhere [33] on Proteomics Service (SCSIE) of the University of Valencia.

#### LC–MS/MS

Spectral peptide library was obtained by liquid chromatography and tandem mass spectrometry (LC–MS/MS) analysis, operating the instrument in a data-dependent acquisition mode. Peptide mixtures were loaded onto a trap column (3 µ C18-CL, 350 μm × 0.5 mm; Eksigent) and desalted with 0.1% TFA at 5 µl/min for 5 min. Peptides were then loaded onto an analytical column (3 µ C18-CL 120 Ᾰ, 0.075 × 150 mm; Eksigent) equilibrated in 5% acetonitrile 0.1% FA (formic acid). Elution was carried out with a linear gradient of 7 a 40% buffer B in A (A: 0.1% FA; B: 5% acetonitrile (ACN), 0.1% FA) at a flow rate of 300 nL/min over 60 min. Eluted peptides were analyzed in a mass spectrometer nanoESI-qQTOF (TripleTOF 6600; SCIEX). Up to 100 ions were selected for fragmentation after each survey scan. Data files were processed using the ProteinPilot search engine (version 5.0, SCIEX) to search the Swiss-Prot database with the following parameters: Trypsin specificity, IAM cys-alkylation, Species *Homo sapiens*, and the search effort set to through and FDR correction.

Quantitative analysis of individual samples was performed by sequential window acquisition of all theoretical spectra-mass spectrometry (SWATH-MS). Peptides were analyzed by LC using a trap column and analytical column as previously described but operating the TripleTOF 6600 mass spectrometer instrument in SWATH mode. We used 100 variable windows from 400 to 1250 m/z with a total cycle time of 2.79 s. Quantitative data was extracted from.wiff files with Peak View 2.2 (SCIEX) using the peptide library generated by ProteinPilot as indicated above. For every protein in the spectral library, a maximum of 20 peptides were quantified among those with a 95% confidence threshold and an FDR lower than 1%. Shared peptides were excluded. For every peptide, a maximum of 6 transitions (fragment ions) were quantified. All proteins contained in the library were monitored in all samples, producing complete quantitative matrices.

#### Data analysis

SWATH quantitative data (protein areas) were median normalized and Log2 transformed. Principal component analysis (PCA) was performed with the Orange data mining toolkit to inspect sample grouping and similarity. Differentially expressed proteins were selected by Student t-test using a *p*-value of 0.05. Functional analysis of differential proteins was performed using STRING database [34] and Cytoscape StringApp [35] within Cystoscape software [36].

### Proteotranscriptomic data integration

Differential proteins identified by RNA-seq and proteomics were pooled in a single protein list with unique UniProt accession codes. Functional analysis of differential proteins was performed using STRING database [34] and Cytoscape StringApp [35] within Cystoscape software [36]. To select the most relevant interactions, only high (score 0.7) and highest (score 0.9) confidence interactions were used for oxaliplatin and palbociclib datasets, respectively. A large network of interacting proteins was observed in each dataset containing about 30 (oxaliplatin) and 45 (palbociclib) % of proteins. The networks were further characterized by performing a functional enrichment analysis of the main modules observed. In the case of palbociclib dataset, a part of the network, containing mainly metabolic proteins, has a more diffuse appearance. In order to gain some information regarding the main metabolic pathways included in this part of the network, their proteins were clustered using the MCL algorithm within StringApp using a Granularity parameter of 2. A functional enrichment analysis was performed on the resulting clusters containing 4 or more proteins. In addition, the metabolic proteins were mapped into KEGG Metabolic pathways (hsa01100).

### Western blot analysis

To verify some of the differentially expressed proteins only detected by proteomics, we performed a Western Blot analysis. We followed a previously published protocol [37]. Briefly, whole-cell protein extracts were prepared using RIPA buffer (50 mmol/L Tris–HCl pH 7.5, 150 mmol/L NaCl, 0.1% SDS, 1% Triton x-100, 0.5% deoxycholic acid sodium salt (w/v)) supplemented with 2 μL/mL protease inhibitor cocktail (Sigma) and 10 μL/mL phosphatase inhibitor cocktail (Sigma). Samples were sonicated and centrifuged at 14,000 × g speed for 20 min at 4 °C. The protein concentration was quantified by BCA protein assay kit (Thermo Scientific). 20ug of total protein were loaded per well on 12% SDS-PAGE, transferred onto a nitrocellulose membrane and incubated with the following primary antibodies from Santa Cruz: anti-TCP1 (Cat. No. sc-374088), anti-CCT2 (Cat. No. sc-374152) and anti-CCT8 (Cat. No. sc-377261). Immunoreactive bands were recognized using peroxidase-conjugated secondary antibody (DAKO). Immunoblots were visualized using the “ECL Western Blotting detection kit” reagent (GE Healthcare) and the ImageQuant LAAS 400 (Healthcare Bio-Sciences) system. Quantification of detected bands was carried out using ImageJ.

### Drug assays

PDOs were trypsinized till single cells, plated in 50% BME domes with complete medium in 96-well plates and 48 h later, after having observed the formation of organoids, treated with increasing doses of different drugs. 5-Fluorouracil and oxaliplatin were kindly provided by HCUV Pharmacy. The following drugs were purchased from Selleck: SN-38 (cat. No. S4908), erlotinib (cat. No. S7786), trametinib (cat. No. S2673), tepotinib (cat. No. S7067), erdafitinib (cat. No. S8401), TAS-102 (cat. No. S8539), alpelisib (cat. No. S2814), palbociclib (cat. No. S1579), olaparib (cat. No. S1060), GSK458 (cat. No. S2658), birabresib (cat. No. S7360), AZD6738 (cat. No. S7693), regorafenib (cat. No. S1178). After 120 h of treatments, CellTiterGlo 3D (Promega, cat. No. G9681) viability assay was performed following the manufacturer’s instructions. All experiments were performed with three technical replicates and with at least two independent biological experiments. Area under the curve (AUC) was calculated with trapezoid rule. Statistical analysis and graphical representation were performed with GraphPad Prism software v. 8.2.1. Experimental replicates of drug screening were compared with one-way ANOVA assuming a *p* < 0.05 as statistically significant and represented as linear regression. For each drug a pattern was assembled containing the corresponding Ln-AUCs across the treated organoids. Next, Z-scores were calculated for each drug, considering median Ln-AUC and standard deviation for each point (LnAUC-mean/standard deviation). Finally, the patterns were aggregated column-wise into a matrix. The obtained matrix was used to assess the relative sensitivity/resistance of each organoid line.

## Results

### PDOs can be established from pre-treated advanced colorectal cancer patient samples

A total of 34 patients with advanced CRC were included in the present study between November 2018 and November 2020, and 50 samples were collected, mainly from liver metastasis. The primary tumor was biopsied only when metastases were not accessible. In the presence of more than one liver metastasis, a sample was taken from each one. We included both naïve and heavily pre-treated patients. Organoids’ growth was observed in 29 lines from 22 patients, with an overall success rate of 59%. All sample characteristics are shown in Supplementary Table S1. Most biopsies were performed at surgery. PDOs establishment was successful in 90% of cases without chemotherapy during the previous 6 months, dropping to 51% if preoperative chemotherapy had been administered, although the difference was not statistically significant. The PDOs’ establishment rate was not affected by primary tumor location (PTL, left vs. right vs. rectum), tissue biopsy site (primary vs. metastasis), or *RAS/RAF* mutational status. In 19 samples from 12 patients no organoids could be grown, this culture failure owing mainly to lack of initial growth, arrested growth or initial contamination. We observed heterogeneous growth behavior among our PDOs. Some could be established as long-term cultures (more than 3 months in culture and over 10 passages) while some others could only grow as short-term cultures (1–3 months in culture and 1–9 passages) [38] (Supplementary Fig. S1). Long-term cultures displayed an exceptional capacity for multiple freeze thaw cycles. No specific molecular features were identified as predicting long-term versus short-term culture establishment.

### PDOs recapitulate morphology and immunohistochemistry and could be derived from tissues with low cellularity

**Fig. 1:**
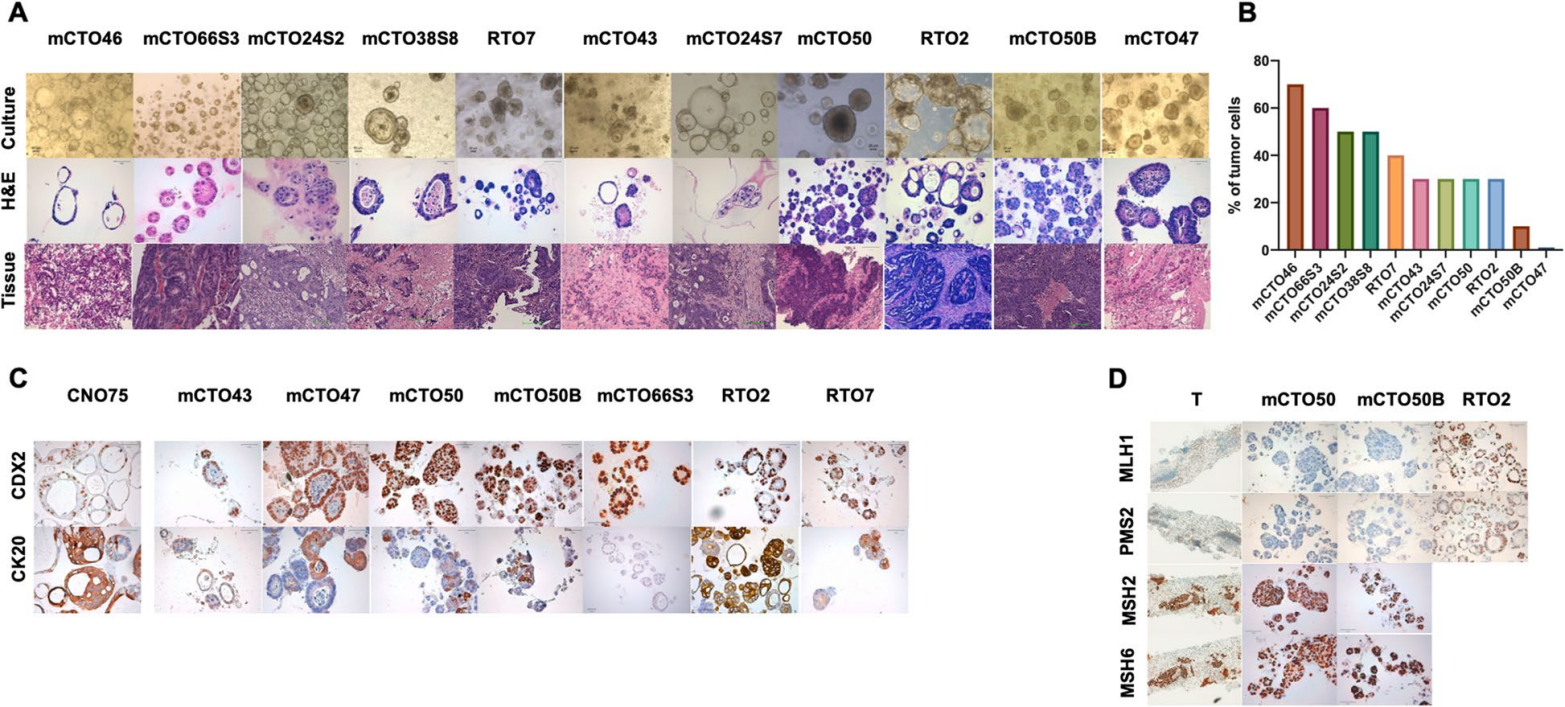
PDOs recapitulate morphologic features of original tissue and can be obtained from low cellularity biopsies. (**A**) Culture images of some PDOs lines (top panel; scale bar 50 μm, 20X), compared with corresponding H&E staining (hematoxylin and eosin) (middle panel; scale bar 50 μm, 20X) and with matched tissue stained with H&E (bottom panel; scale bar 100 μm, 10X). (**B**) Corresponding percentage of tumor cells assessed as percentage of neoplastic cells with respect to the total amount of viable cells in the tissue sample from which the culture was derived, assessed by the pathologist. (**C**) IHC for CDX2 and CK20 antibodies in a selection of PDOs. CNO75 is a normal mucosa organoid from a CRC patient, used as control for both markers. (Scale bar 50 μm, 20X). (**D**) Lack of expression of MLH1 and PMS2 in a core biopsy from a liver metastasis of patient 50 and its corresponding derived PDOs. RTO2 is a pMMR model used as positive control. (Scale bar 50 μm, 20X). T: tissue

PDOs were stained with hematoxylin and eosin (H&E) to show cellular architecture, which faithfully reproduced the morphology seen in culture (Fig. 1A, top and middle panel). Cellular architecture presents similarities with corresponding tissues (Fig. 1A, bottom panel).

To elucidate whether tumor cell percentage could impact on the capacity to obtain organoids, each tissue section destined for organoid culture was revised by a dedicated pathologist. A cut-off of 30% tumor cells was applied to classify our samples. Our data indicate that PDOs can be grown independently of tumor cell proportion found in the original tissue (two-tailed Fisher’s exact test *p* = 0.4568, Fig. 1B).

IHC staining shows that PDOs are positive for CDX2 and CK20, intestinal markers that are both employed in standard diagnosis of CRC (Fig. 1C). The only exception was seen in mCTO66S3, which tested CK20 negative. The original tissue displayed weak and parceled expression, with most cells clearly negative (Supplementary Fig. S2). Indeed, loss of CK20 expression may be considered a negative prognostic marker in some settings [39]. CDX2 and CK20 expression of corresponding tissues is showed in Supplementary Fig. S2, where a weak expression of CK20 and no CDX2 staining in mCTT47 was observed, probably due to low cellularity, high level of fibrosis and intratumoral heterogeneity. On the other hand, our PDO mCTO50 and mCTO50B, derived from a patient with MSI high showed lack of expression of MLH1 and PMS2 proteins, as was seen in the original tissue (Fig. 1D, Supplementary Fig. S2).

### PDOs recapitulate the genomic and transcriptomic profile of the original tumor

To genomically compare organoids and their original tissues, we employed NGS analysis using a customized panel [19]. A high correlation was found regarding germline variants, single nucleotide variants (SNVs), short insertions and deletions (*r* > 0.8, *p* < 0.0001, Supplementary Fig. S3A-B). The percentage of tumor cells, necrosis, mucinous proportion, and tumor stroma ratio were calculated by a dedicated pathologist. These indices did not interfere with the ability of PDOs to reproduce the original tissue genomic profile (Supplementary Fig. S3C), except when low cellularity was present (Supplementary Fig. S3B).

**Fig. 2:**
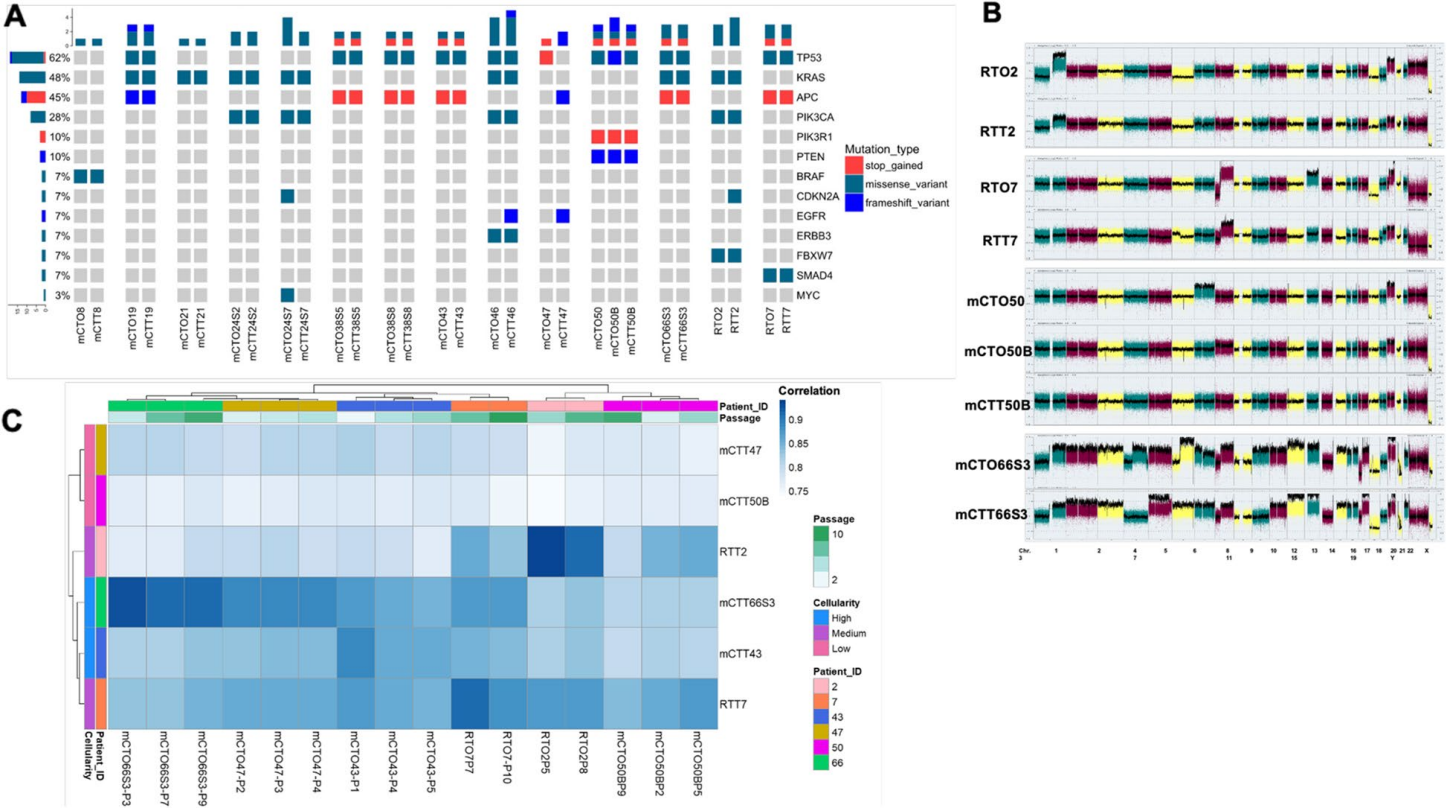
PDOs recapitulate genomic and transcriptomic profile of original tissues. (**A**) Concordance of SNVs and InDels between PDOs and corresponding tissues. On the left percentage of genomic alterations detected across the study subjects. (**B**) Whole Genome View representation of long-term PDOs and corresponding tissues according to Cytoscan HD. Data is expressed as the weighted log2 ratio of the copy number on the left Y-axis, and the chromosome number on the X-axis. 0 corresponds to diploid, upper and lower spikes indicate gain and loss regions, respectively. mCTO50 tissue was not available. (**C**) Heatmap showing the Pearson correlation coefficient (color key) between tumors (rows) and organoids (columns) based on the normalized counts as described in materials and methods section. Dendrograms show the hierarchical clustering based on the complete method and Euclidean pair-wise distance. Different passages from the same organoid culture are included

As expected, the most prevalent mutations affected *TP53*, *KRAS* and *APC* (Fig. 2A), and besides these, *PIK3CA*, *PTEN* and *SMAD4* mutations were also detected, encompassing all relevant mutations in CRC. The main driver oncogenic mutations and hotspot variants are represented in Table 1. In most cases, allelic fraction (AF) was higher in PDOs (paired *t* test, *p* < 0.0001), reflecting their enrichment in epithelial cells. Moreover, CytoScan HD SNP-array was conducted on PDOs and matched fresh tissue to detect copy number alterations and LOH. Likewise, PDOs recapitulate the overall copy number variation profile, and are significantly enriched (Fig. 2B). CytoScan results were validated by ddPCR (Supplementary Fig. S3D).

**Table 1:** Allelic fraction (AF) distribution in PDOs and matched tissues of main driver, hotspot and not hotspot mutations with potential relevance. Each mutation has been manually reviewed in Integrative Genome Viewer (IGV). Only mutations with an AF of at least 5% in PDOs have been included

Patient	Gene	Mutation	PDOs VAF	Tissue VAF
mCTO8	BRAF	p.D594G	0.5118	0.3675
mCTO19	TP53 KRAS APC	p.R273C p.G12S p.E1309Dfs*4	0.4319 0.3282 0.1862	0.3766 0.356 0.1247
mCTO21	KRAS MYC	p.G12D p.T73P	0.4274 0.0975	0.2 -
mCTO24S2	PIK3CA KRAS APC	p.H1047R p.G12D p.Y935*	0.5056 0.5089 1	0.1642 0.1682 0.285
mCTO24S7	PIK3CA KRAS APC MYC CDKN2A	p.H1047R p.G12D p.Y935* p.T73P p.D74A	0.4852 0.4978 0.9965 0.0975 0.0898	0.1529 0.1935 0.3412 - -
mCTO38S5	TP53 APC FBXW7	p.R248W p.Q1367* p.R222*	0.9967 0.994 0.6828	0.5272 0.4723 0.2438
mCTO38S8	TP53 APC FBXW7	p.R248W p.Q1367* p.R222*	0.9966 0.9933 0.5113	0.2991 0.3066 0.1531
mCTO43	TP53 APC	p.Y236H p.Y935*	0.9975 0.9804	0.3309 0.2581
mCTO46	ERBB3 PIK3CA KRAS TP53	p.T355I p.K111E p.G12S p.R273H	0.6528 0.5521 0.99 0.99	0.502 0.3632 0.4512 0.5022
mCTO47	TP53 MYC GNAS APC EGFR	p.R213* p.T73P p.R201S p.E1379Vfs*7 p.L833Gfs*63	0.9983 0.1158 - - -	- - 0.0606 0.156 0.0895
mCTO50	TP53 PTEN PIK3R1	p.G245S p.K267Rfs*9 p.R348*	0.4752 0.9504 0.4696	na
mCTO50B	TP53 PTEN PIK3R1	p.G245S p.L257V p.K267Rfs*9 p.R348*	0.4893 0.1695 0.9511 0.4993	0.2895 0.0848 0.3198 0.1592
mCTO66S3	TP53 KRAS APC	p.M237I p.G12V p.E1379*	0.988 0.4407 0.998	0.8701 0.4611 0.9554
RTO2	PIK3CA KRAS FBXW7	p.E545K p.G12V p.S582P	0.464 0.5106 0.4626	0.2827 0.3149 0.2964
RTO7	TP53 APC SMAD4	p.C275Y p.Y935* p.P356S	1 0.4842 1	0.2904 0.1005 0.1742

*VAF* Variant allele frequency

RNA-seq was also performed to compare gene expression between PDOs and corresponding tissue and to analyze whether it was stable over time in culture. This is a key issue, considering PDOs as in vitro “avatars” of patient tumors. Three biological replicates deriving from distinct culture passages were employed for each organoid line, except for RTO2 and RTO7 for which two replicates were employed, due to technical issues. Overall, PDOs and tissues cluster separately because of the co-existence of more cell types in tissues and stromal genes (Supplementary Fig. S3E). However, narrowing down the analysis to non-stromal genes contribution (supplementary methods), significant expression correlation can be observed between each PDOs line and its corresponding tissue (Fig. 2C). The two exceptions were mCTO50B and mCTO47, which were derived from a tissue with low cellularity (less than 10%). Interestingly, PDOs from different passages exhibit a similar gene expression profile, indicating that the influence of culture condition is minimal and that this profile is stable over time.

### PDOs show differential anti-tumor response to standard and experimental treatments

To study whether PDOs could be an appropriate model to assess differential response to standard therapies, we exposed our long-term models to several approved drugs. The reproducibility of drug treatment results in PDOs was confirmed across several experiments (Supplementary Fig. S4A). Table 2 summarizes the clinical and molecular characteristics of patients whose models were used.

**Table 2:** Clinical and molecular characteristics of patients from which long-term cultures have been generated

ID sample	M/F	PTL	RAS/RAF	MSI/MSS	Other mutations	Line	PFS (months)	OS (months)
RTO2	F	rectum	*KRAS*	MSS	*PIK3CA*	XELOX	18	nr
RTO7	M	rectum	wt	MSS	*SMAD4*	FOLFIRI cetuximab	3	11
mCTO50 mCTO50B	F	cecum	wt	MSI	*PTEN*	1st FOLFOXIRI beva 2nd pembrolizumab	4 nr	nr
mCTO66S3	M	rectum	*KRAS*	MSS		upfront surgery – > first FOLFOX	NED	nr

*M/F* Male/female, *PTL* Primary tumor location, *wt* Wild type, *PFS* Progression-free survival, *OS* Overall survival, *XELOX* Capecitabine and oxaliplatin, *FOLFIRI 5*-Fluorouracil and irinotecan, *FOLFOX 5*-Fluorouracil and oxaliplatin, *FOLFOXIRI 5*-Fluorouracil oxaliplatin and irinotecan, *nr* Not reached, *NED* No evidence of disease

**Fig. 3:**
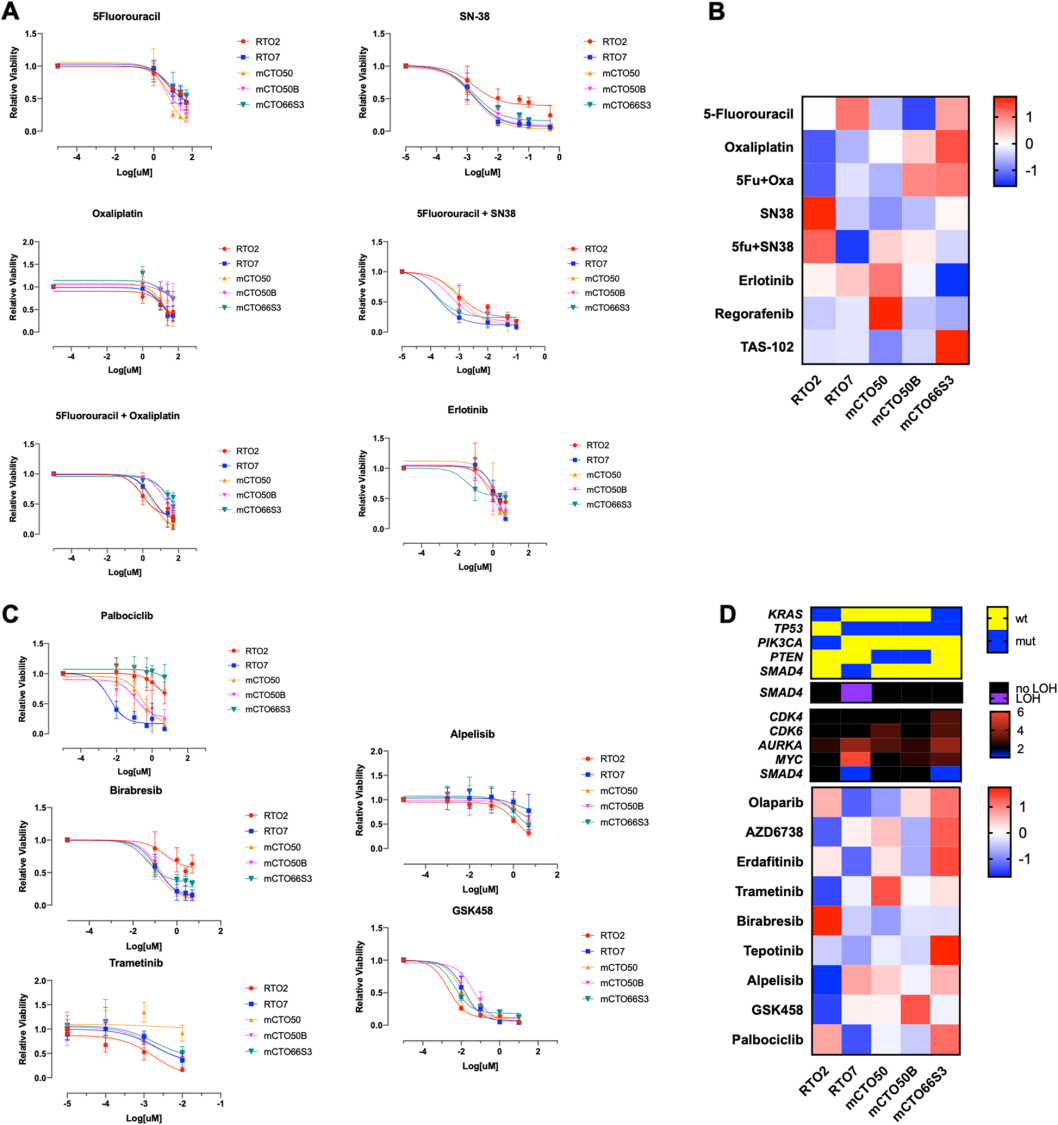
PDOs response to drug screening. (**A**) Log transformed dose–response curves in selected standard drugs and non-standard (**C**) drugs. (**B**) Z-score Ln-AUCs heatmap (red: no response; blue: good response) for standard treatment. (**D**) Z-score Ln-AUCs heatmap (red: no response; blue: good response) for non-standard treatment (lower panel), matched with genomic data. In the upper panel are depicted main gene mutations, in the following loss of heterozygosity (LOH) and in the last copy number variations

All the established PDOs exhibited a heterogeneous response to chemotherapy (Fig. 3A). As an example, mCTO50B (a PDO obtained after chemotherapy) had a worse response than mCTO50, which was generated from the initial tumor before any therapy was given, so the former could represent a potential model of chemo-resistant disease.

SN-38 (the active metabolite of irinotecan) was highly active in vitro, and only RTO2 displayed lower sensitivity compared to the other lines in our panel. Adding oxaliplatin to 5-FU resulted in a general decrease in PDOs viability (Supplementary Fig. S4B). Along these lines, combination with SN-38 seemed to be more effective than single agent exposure (Supplementary Fig. S4B, not statistically significant). Anti-EGFR treatment with erlotinib showed modest activity in all lines, including the *RAS* wild type RTO7 PDO. Regorafenib also showed modest in vitro activity, whereas trifluridine/tipiracil (TAS-102) was more active in all PDOs except mCTO66S3 (Supplementary Fig. S4C). A matrix representation of normalized Z-score Ln-AUCs (Fig. 3B) identifies relative resistance/sensitivity to a single drug among different PDOs.

To further explore sensitivity to other anti-cancer agents, our PDOs were exposed to different drugs not currently included in the standard of care for advanced CRC. Agents targeting pathways altered in our models were selected. Response was matched with genomic alterations in all models (Fig. 3C-D). Differential responses were seen for palbociclib, trametinib, alpelisib, the BET inhibitor birabresib (Fig. 3C). For example, RTO7 showed a dramatic response to palbociclib, a selective inhibitor of CDK4/6 kinases, in comparison with the other PDOs, particularly RTO2 and mCTO66S3. Considering genomic data, the lower responder mCTO66S3 has the higher copy number level of both *CDK4* and *CDK6*, reported as a mechanism of resistance [40]. Additionally, RTO7 has the highest copy number of *c-MYC*, potentially indicating cell cycle activation [41–43]. Moreover, this line has a *SMAD4* p.P356S missense variant, in a hotspot region with LOH, thus explaining the nearly 100% mutant allelic fraction (Table 1). Despite the lack of functional validation, it is predicted to be oncogenic by several databases, leading to a loss-of-function of the protein [44] and is associated with increased c-MYC activity [45]. Indeed, the high response to palbociclib observed in RTO7 certainly warrants further research, as all these data point to a “MYC-addicted” phenotype of RTO7.

The BET inhibitor birabresib (Fig. 3C, middle) showed good activity in all lines, particularly in *SMAD4* loss RTO7 and mCTO66S3 (Fig. 3D, Supplementary Fig. S4D, S5A), confirming synthetic lethality via restoration of MYC inhibition [45]. MEK-inhibitor trametinib showed modest activity in all lines except for *KRAS* mutant RTO2 (Fig. 3C), while PI3Kα-inhibitor alpelisib showed modest activity in all PDOs except for a slightly more in PIK3CA mutant line RTO2 (Fig. 3C, upper right). Intriguingly, the PI3K-mTORC1/2 inhibitor GSK458 was significantly active, not only in *PIK3CA* mutant RTO2, but also in mCTO66S3 (Fig. 3C, lower right). In contrast, mCTO50B had a significantly worse response. Indeed, this PDO has a frameshift mutation of the *PTEN* gene (p.K267Rfs*9) reported as likely oncogenic in OncoKB™, and we showed that it leads to reduced gene expression and a loss of PTEN expression per IHC (Supplementary Fig. S5B). We also performed additional drug testing without observing relevant activity (Supplementary Fig. S4D).

### PDOs differentially replicate patient response depending on the site of disease

**Fig. 4:**
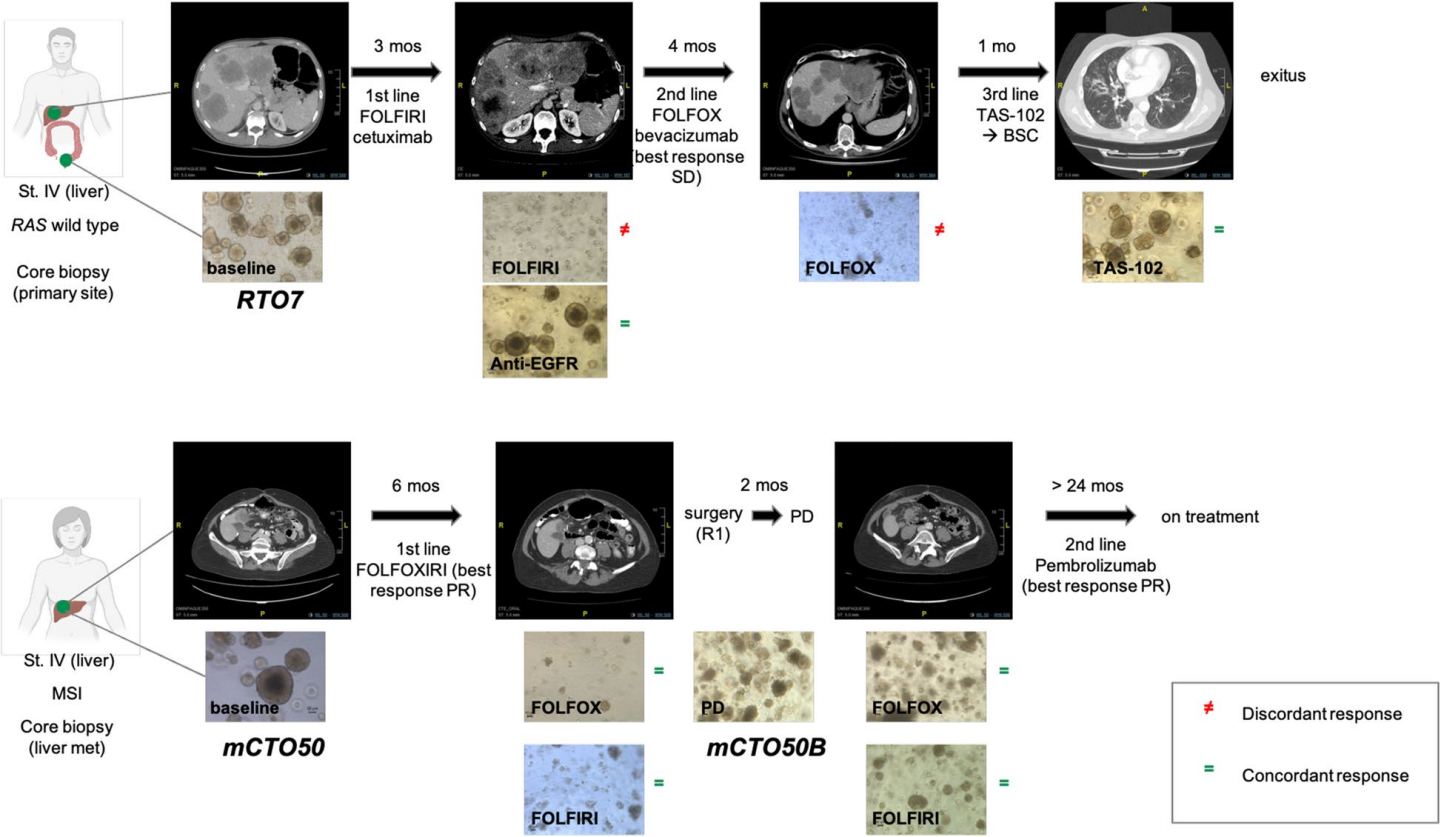
Patient’s response matched with PDOs drug assays, RTO7 in the upper panel and mCTO50 in the lower panel. Each CT scan response evaluation is compared with corresponding treatment administered in the corresponding PDOs. ≠ means discordant response, = means concordant response. Mo(s): months in terms of progression-free survival; St: stage; FOLFIRI: 5-fluorouracil and irinotecan; FOLFOX: 5-fluorouracil and oxaliplatin; FOLFOXIRI: 5-fluorouracil, oxaliplatin and irinotecan; BSC: best supportive care; SD: stable disease; PR: partial response; PD: progressive disease; R1: microscopic residual tumor

To determine whether our PDOs would also reproduce patient response to treatment, we retrieved clinical information and matched drug response observed for mCTO50 and RTO7 (Fig. 4), in which follow up data events were available. mCTO50 was generated from liver metastasis of a patient with MSI-high CRC before initiating any anti-tumor therapy. This patient received cytoreductive chemotherapy with FOLFOXIRI (a combination of 5-FU, leucovorin, oxaliplatin and irinotecan), presenting appropriate tumor shrinkage and allowing liver metastases resection. When tumor response was tested in vitro, mCTO50 was significantly more sensitive to 5-FU and oxaliplatin than mCTO50B, which was generated from the remaining residual disease tissue obtained at surgery. The drug treatment effect in this PDO was comparable to that observed in the patient as mCTO50. The patient had disease progression shortly after surgery, indicating that chemotherapy failed to exert a long-term effect. A similar degree of sensitivity to irinotecan was observed in both PDOs, however, prompting us to hypothesize that resistance mechanisms to this drug were not yet established.

RTO7 exhibited a good response to irinotecan-based chemotherapy, received as part of the patient’s first line treatment regimen (Table 2). Conversely, patient response was poor (showing progression at first CT scan). The only concordant response observed was for the anti-EGFR agent and for TAS-102 (i.e. lack of response). However, it should be taken into consideration that the organoid line derived from the primary location rather than liver metastases, which could not be biopsied. This highlights the intrinsic biological heterogeneity of CRC and the need to biopsy metastatic sites in the context of translational studies.

### Proteotranscriptomic analysis in PDOs in relation to oxaliplatin or palbociclib sensitivity

Our aim was to uncover functional correlates between baseline proteotranscriptomic expression and drug sensitivity in PDOs. To assess the steady-state protein abundance, a SWATH-MS based label-free quantitative proteomics analysis was performed as a more reproducible strategy in a translational context. The relative level of the 1157 proteins quantified in all samples (FDR < 1%) helped us to distinguish each PDO by principal component analysis (PCA) (Supplementary Fig. S5C) and these were consistently reproduced at different culture passages. We focused on understanding the molecular mechanisms involved in sensitivity to palbociclib and the lack of response to oxaliplatin, where the most striking differences were observed among PDOs. Three biological replicates proceeding from different culture passages were used for each PDOs line.

**Fig. 5:**
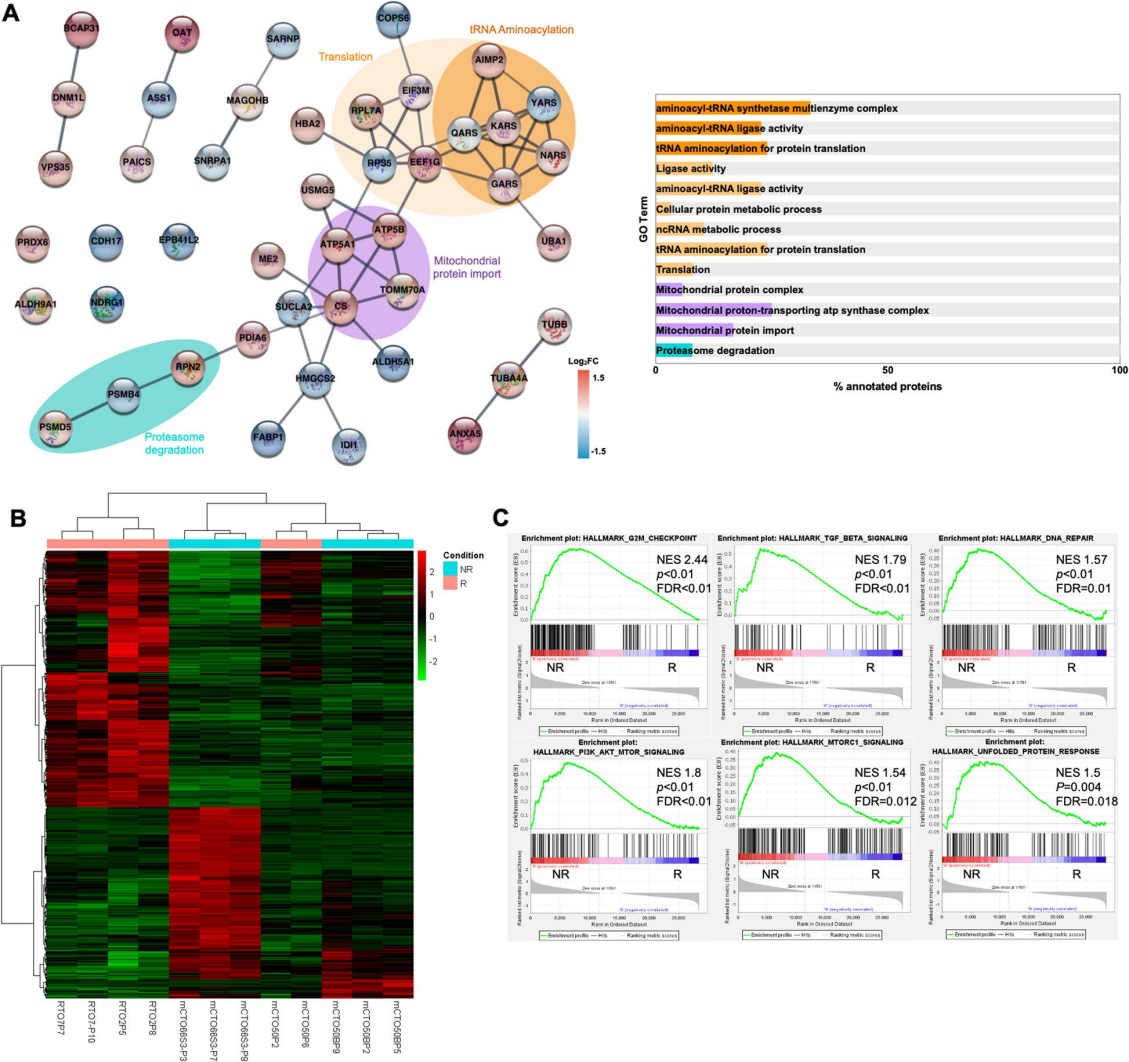
Proteotranscriptomic characterization of oxaliplatin responder lines in comparison with no-responder ones. (**A**) Network mapping of 95 proteins showing the 4 sub-clusters composed of more than 3 proteins. Relevant proteins with no associations to others are represented as isolated nodes. Colors are depicted according to the protein abundance (log2ratio) compared to responder PDOs (left panel). Bar-chart of GO terms represented as percentage of annotated proteins using the same color coding (right panel). (**B**) Hierarchical clustering heatmap of differentially expressed genes per transcriptomic. (**C**) GSEA hallmarks analysis of RNA-seq data. NES: normalized enrichment score. FDR: false discovery rate. R: responder; NR: non responder

RTO2, RTO7 and mCTO50 showed better response to oxaliplatin than mCTO50B and mCTO66S3 (unpaired t-test *p* = 0.0038) (Fig. 3A). A proteomics analysis (Supplementary file S1) identified 95 differentially expressed proteins (51 up-regulated, 44 down-regulated) in non-responder compared to responder organoids. Among the proteins identified with the greatest positive and negative fold change (Fig. 5A), we found several previously associated with oxaliplatin resistance. As an example, resistant PDOs showed higher levels of PRDX6, which is a negative regulator of ferroptotic cell death [46], a process that enhances CRC sensitivity to oxaliplatin [47]. Another up-regulated protein was ALDH9A1, a member of the ALDH family of proteins which are involved in aldehyde detoxification which in turn is associated with acquired chemoresistance in colorectal cancer cells [48]. These same models exhibited lower levels of NDRG1, a replication stress response protein which can inhibit epithelial-mesenchymal transition (EMT), a process that has been associated with phenotypes of chemoresistance [49], and of CDH17, which has been reported as a marker of good response to 5-fluorouracil and oxaliplatin-based chemotherapy [50].

To gain insight into alternative mechanisms of oxaliplatin resistance, a functional protein–protein interaction network of differentially expressed proteins was evaluated by STRING database (http://string-db.org, version 11). Using a high confidence score, some interesting interactions were identified, with some subclusters remaining at the highest confidence score (Fig. 5A). Gene ontology (GO) analysis revealed an increase of proteins related with translation in non-responder models, with an interesting subcluster enriched in tRNA-aminoacylation process that included different aminoacyl-tRNA synthetase (ARSs) and an auxiliary protein AIMP2, joined to form the cytoplasmic multi-tRNA synthetase complex [MSC] [51]. Beyond their role in protein synthesis, ARSs were related to the induction of unfolded protein response, which in turn was associated with escape from chemotherapy induced senescence [52].

Another enriched process in non-responder models was mitochondrial import, including among other proteins two subunits of the ATP synthase (ATP5A and ATP5B), a mitochondrial membrane protein complex mediating ATP synthesis, and citrate synthase (CS), one of the key enzymes in the tricarboxylic acid cycle (TCA), indicating a switch from a glycolytic based to a mitochondrial metabolism with oxidative phosphorylation (OXPHOS) as main source of energy. This could represent a way for some cancer cells to repair platinum-induced DNA damage more efficiently [53].

In the RNA-seq analysis, 597 differentially expressed genes were detected (Fig. 5B). GSEA study showed that non-responding PDOs were enriched in G2M checkpoint, TGFbeta and DNA repair hallmarks (Fig. 5C), somewhat expected as regards oxaliplatin response. Indeed, it acts by inducing DNA adducts, therefore an increase in the capacity to repair damaged DNA could help the cancer cell to survive. A key moment of DNA repair occurs during G2 to M phase transition. In addition, TGFbeta contributes to oxaliplatin resistance in CRC [54]. We noted that non-responder PDOs showed enriched unfolded protein response and PI3K-Akt-mTOR and mTORC1 signaling hallmarks (Fig. 5C). As previously indicated, the former could be involved in the escape from chemotherapy induced senescence [58].

**Fig. 6:**
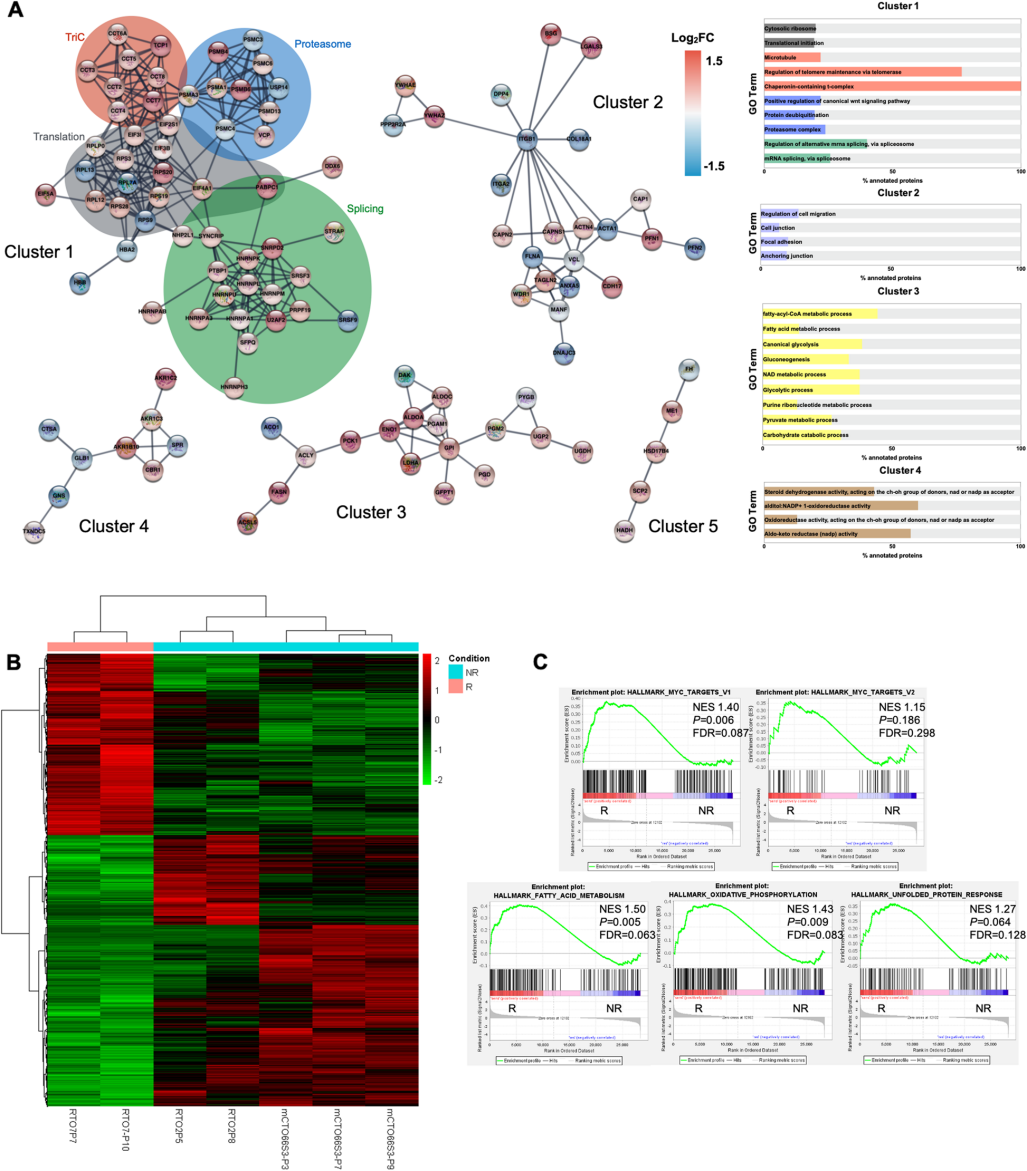
Proteotranscriptomic characterization of Palbociclib responder line in comparison with no-responder ones. (**A**) Network mapping of 111 proteins showing the 4 clusters composed of more than 3 proteins. Proteins with no associations to others were removed and nodes are colored according to the protein abundance (log2ratio) compared to responder PDO (left panel). Bar-chart of GO terms represented as percentage of annotated proteins using the same color coding (right panel). (**B**) Hierarchical clustering heatmap of differentially expressed genes per transcriptomic. (**C**) GSEA hallmarks analysis of RNA-seq data. NES: normalized enrichment score. FDR: false discovery rate. R: responder; NR: non responder

After observing high sensitivity to palbociclib in RTO7 (unpaired t-test *p* = 0.0002), and to understand the molecular mechanisms involved in this, a comparison of protein expression profiles was made, revealing 245 significantly changing proteins in RTO7 versus non-responder models (RTO2 and mCTO66S3) (Supplementary file S1). Functional protein–protein interaction networks were evaluated using the STRING database with a highest confident score (Fig. 6A). Gene ontology (GO) functional enrichment analysis matched the largest identified cluster (55 of 111 proteins) to biological processes related to protein synthesis, folding and degradation, and with mRNA splicing process via spliceosome, with most of the proteins upregulated in our RTO7 PDO as a palbociclib- responder model (Supplementary Fig. S5). We identified a subcluster comprising all the eight proteins that form the T-complex protein Ring Complex (TRiC) (Supplementary Fig. S5D), an essential eukaryotic molecular chaperonin that aids in the folding of ~ 10% of the proteome including oncoproteins [55].

Other top processes included in the remaining clusters were related to regulation of cellular component organization, cell adhesion, and metabolic processes, among others (Fig. 6A). All these processes point towards a high proliferative phenotype supported by the up regulation of different mechanisms that preserve proteome integrity.

Transcriptional-wide changes were also observed in palbociclib sensitive model (Fig. 6B). GSEA analysis showed that RTO7 is enriched in MYC targets hallmarks, fatty acids and lipid biosynthesis (which together are a consequence of MYC activation [56]) and unfolded protein response (Fig. 6C), a process that indicates an alteration of protein homeostasis. In the latter hallmark, *NPM1*, a c-MYC activator [57], is one of the most significantly enriched genes. We could not quantify Myc protein, by SWATH-MS analysis, maybe due to relative scarcity being it a transcription factor. Nevertheless, we were able to detect higher levels of NPM1 among non-clustering proteins.

We observed a weak correlation between protein abundance and transcript quantification among differentially expressed proteins in both comparisons (palbociclib: *r* = 0.1477, *p* < 0.01; oxaliplatin: *r* = 0.1446, *p* = 0.02; Supplementary Fig. S6A), indicating the occurrence of complex post-transcriptional regulation. For instance, the relevant proteomic data regarding TRiC and ARSs roles in palbociclib responder and oxaliplatin non-responders’ models respectively, were not confirmed by RNA-seq (Supplementary Figure S6B). Nevertheless, some relevant processes related to protein folding, biosynthesis and proliferative features were captured by both RNA and protein analysis.

### Integrative functional network (IFNA) analysis of proteotranscriptomic data

To gain deeper insight into the processes involved in the differential drug response in PDOs we performed an integrative analytical approach [58]. Thus, we fused the transcriptomics and proteomics differentially expressed datasets and extracted the common functional context through an integrative network analysis by STRING application via Cytoscape.

**Fig. 7:**
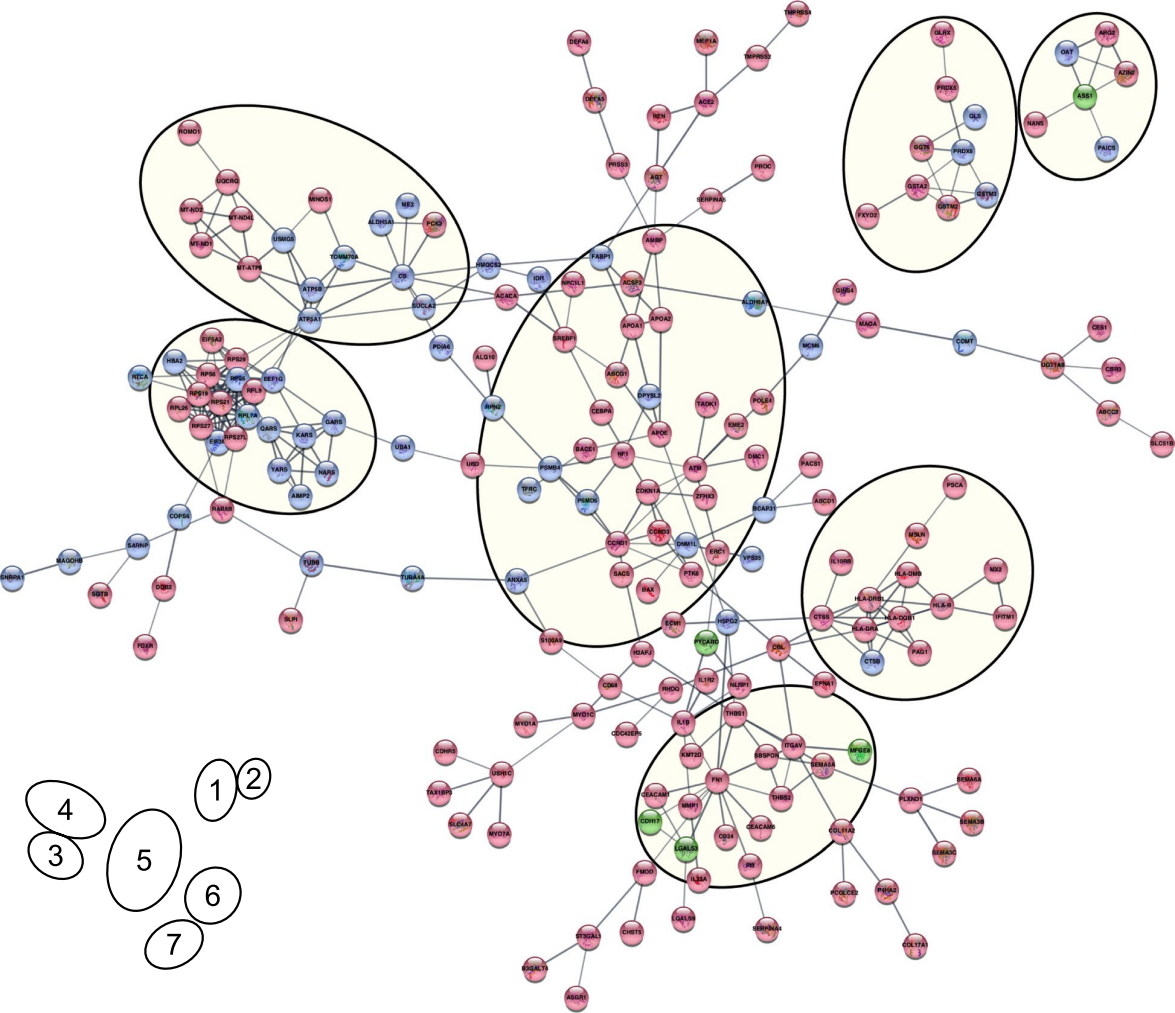
Integrative functional network analysis of oxaliplatin RNA/protein fused dataset. High confidence interactions (score 0.7) showing 7 modules. Nodes in red correspond to proteins identified as differentially expressed by RNA-seq only, in blue those identified by proteomics only and in green those identified by both omics. Key for modules: 1: Glutathione metabolism & antioxidant activity; 2: Nitrogen compound metabolism; 3: Translation & tRNA aminoacylation; 4: TCA & Oxidative phosphorylation; 5: Cell cycle regulation; 6: Antigen presentation; 7: Cell adhesion

**Fig. 8:**
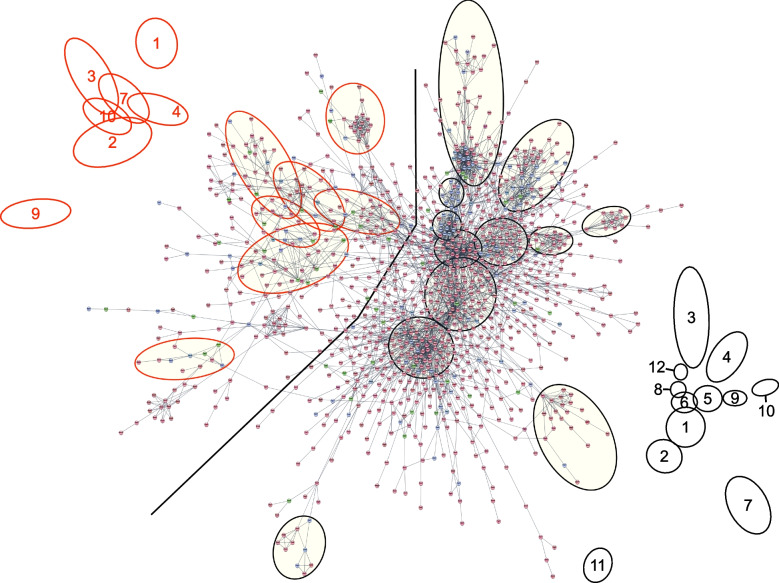
Integrative functional network analysis of palbociclib RNA/protein fused dataset. Highest confidence interactions (score 0.9) showing modules. Nodes in red correspond to proteins identified as differentially expressed by RNA-seq only, in blue those identified by proteomics only and in green those identified by both omics. Grey line marks on the left 7 metabolism related modules with the highest integration. Key for metabolic modules: 1: Oxidative phosphorylation; 2: Pentose phosphate pathway, and Glycolysis; 3: Metabolism of nucleotides, and purinergic nucleotide receptor signaling pathway; 4: PPARA signaling pathway; 7: Lipid biosynthetic process; 9: Oxidoreductase activity; 10: Organonitrogen compound metabolic process. Key for modules on the right: 1: Regulation of signal transduction; 2: Regulation of cell communication and motility; 3: Translation; 4: RNA splicing; 5: Transcription and DNA repair; 6: Cell cycle; 7: Membrane trafficking; 8: Proteasome; 9: Protein ubiquitination; 10: Interferon signaling; 11: ER-Golgi vesicle-mediated transport; 12: TRiC/CCT complex. Details of the modules are indicated in Additional file 2

Data fusion and integrative functional network analysis (IFNA) were conducted for both oxaliplatin and palbociclib drug response comparisons. The functional clusters shown in the integrative network, representing a high level of interaction and integration of the proteomic and transcriptomic data, are referred as modules in the rest of the manuscript. High confidence interacting proteins are shown in Fig. 7 and Fig. 8, where relevant modules are marked with a shading. In both cases, large networks containing approximately 30–45% of the differential proteins were observed. Interestingly, most of the proteomic clusters and RNA-seq hallmarks (Fig. 5B and Fig. 6B) are now contained in the networks, including some previously isolated clusters. The functional enrichment analysis of the different modules observed in IFNA are listed in Additional File 2 for oxaliplatin and palbociclib comparisons.

In the context of oxaliplatin response, the whole dataset contained 12 proteins commonly represented by both omics, 83 only by proteomics and the remaining by RNA-seq. Only high confidence interactions are shown in Fig. 7 containing the main network and two isolated clusters. Globally it contained 191 proteins, 50 of them were detected only by protein, 136 only by RNA while 5 were commonly detected.

Within the main network, 5 modules were selected to perform a functional enrichment analysis that confirmed the processes highlighted in individual omics profiles and now appear connected into this new integrative network. For instance, the relevant translation and tRNA aminoacylation processes appear in module 3 which is now complemented with RNA data. Another interesting finding is module 4, where the proteomic cluster mitochondria protein import is now complemented with TCA cycle and Oxidative phosphorylation processes by RNA data. Proteasome proteomic cluster and DNA repair RNA-seq hallmark are now enriched processes in module 5.

Some previously unconnected differential proteins now appear integrated into the network. Such is the case of CDH17 in module 7, and, interestingly, PRDX6 that is now part of module 1 enriched in redox metabolism, which may be involved in oxaliplatin resistance.

Regarding palbociclib sensitivity, the fused dataset contained 172 proteins detected by proteomics, 73 detected by both proteomic and RNA-seq, and the remaining detected only by RNA-seq. Figure 8 shows the large network of the highest confidence interacting proteins in palbociclib comparison. Certain modules, related to splicing, translation, proteostasis and metabolism showed a high complementarity between RNA-seq and proteomics. This complementarity reinforces the functional enrichment already defined. Interestingly, TRiC complex, that was only detected as differential at protein level (some of their subunits verified by western blot analysis, Supplementary Fig. S5E), is now complemented by BAG5, involved in endoplasmic reticulum stress regulation and unfolded protein response activation [59]. In fact, it has been described that the protein BAG5 is a co-chaperone involved in protein folding [60].

A new central module related to cell cycle and mainly represented by RNA-seq data, appeared highly connected with the proteasome, TRiC, and (to a lesser extent) with splicing processes. Moreover, the genes that define the GSEA Myc targets signature, a hallmark enriched by RNA-seq analysis, map into this network encompassing all these processes.

The large diffuse metabolism module constitutes a good example of differential RNA and protein integration. Applying a clustering algorithm as described in Methods section, we found a clear enrichment in the pentose phosphates and glycolysis pathways, and also in lipid and nucleotide metabolism in relation to palbociclib sensitivity. When we mapped differentially expressed metabolic genes into KEGG Metabolic pathway map, we observed a nice complementation of both omics (Supplementary Figure S7). These cellular pathways can satisfy a high energy and anabolic precursors demand to sustain enhanced growth and proliferation in this putative Myc-addictive phenotype.

## Discussion

Advanced CRC is a complex and heterogeneous disease in which resistance development limits the success of drug treatments. PDOs have been shown to maintain the same genomic and phenotypic features of original tumor tissue and can be used for high-throughput drug screening, enabling us to anticipate clinical response. Although the number of patients included in this study is not high, we demonstrate the potential of integrating PDO drug response with a deep proteotranscriptomic analysis, providing novel insights into sensitivity to oxaliplatin and palbociclib.

We report the generation and characterization of a library of PDOs from advanced CRC patients, including both naïve and pretreated subjects, showing that it is possible to obtain organoids even from heavily pretreated samples. We were able to generate a basal and progressive MSI-high PDO, to our knowledge the first study to achieve these results. MSI lines were generally derived for primary CRC tumors [11].

Our organoids recapitulate their matched tumor tissue at genomic and transcriptomic level. We observed an increase in PDO variant allele frequencies (VAFs) and copy number variations compared to corresponding tissues, due to the enriched epithelial nature of organoids, avoiding the dilution effect of the DNA of other cell types such as fibroblast, endothelial and immune cells, etc. that are present in the tumor microenvironment (TME). Therefore, we could detect rare genomic events, such as the previously characterized *SMAD4* and *PTEN* mutations (Table 1), uncovering their potential oncogenic relevance (*PTEN* mutation, for instance, leads to protein expression loss). A few reports have been published on the comparison of transcriptomic similarity between PDOs and original tissues [61] and direct comparison showed a clear difference between organoids and tissues, as expected. Nevertheless, after adjusting for stromal gene contribution, organoids displayed a highly similar expression profile compared with that of tissue, which is stable over time. This proves an only minimal effect of culture condition, and that expression findings are robust and result from cancer cells.

Regarding tissue amount required for organoid generation, we demonstrated the feasibility of deriving reliable cultures even from tissues with a very low cellularity, although the genomic and phenotypic features of these PDOs may show lower correlation with the original tissue. Demonstrating that these models could reproduce and therefore predict patient outcomes is crucial for their use in precision medicine. Indeed, our mCTO50B PDO derived from extremely low cellularity tissue could effectively replicate patient response. Biopsy site could be viewed as a more important issue. Given that not all lesions are easily accessible for biopsy, the use of a primary site as a substitute should be considered with caution as it might not replicate ex vivo the response observed in the clinic. This was the case with RTO7, which could only be used a primary site biopsy, while the response observed in the PDOs failed to perfectly match what it was observed in liver metastasis.

We captured heterogeneous drug response to several agents and performed a proteotranscriptomic analysis of responder and non-responder models to identify baseline molecular features associated with response. This is particularly important for backbone chemotherapeutic agents such as oxaliplatin, for which no biomarkers have been brought into clinical use yet [62]. SWATH-MS was selected for proteomic study as an emerging strategy for biomarker discovery due to the coverage achieved, the reliable quantitation obtained and the possibility to re-interrogate the data [63]. Few studies have characterized organoids at the quantitative proteomic level. We report on the use of SWATH in PDOs in this regard, underlining its potential utility to identify a *bona fide* biomarker of response to anticancer agents, in contrast to most current efforts focused on RNA signatures. Indeed, when individually analyzed, we observed cases where both techniques go in the same direction as well as discrepancies between protein abundance and their corresponding RNAs, probably due to mRNA post-transcriptional regulation. However, transcriptomic experiments have a much wider genome coverage than proteomics. So, we consider that both methodologies are complementary and that a multi-omics approach can provide richer biological information for a better comprehension of the complex pathological molecular mechanisms involved in drug response. In addition, after fusing RNA and protein data into a unique dataset and thanks to IFNA, we could confirm those processes which were detected at RNA or protein level and found new modules, enriched in processes which could have a putative role in differential drug response and that were highlighted after dataset integration.

Some of the differential proteins identified in relation to oxaliplatin response have been previously associated with resistance, but surprisingly, functional enrichment analysis highlights the t-RNA aminoacylation process, including some aminoacyl-tRNA synthetases (ARSs) and an auxiliary protein AIMP2 which forms the MSC complex [51]. ARSs are traditionally considered housekeeping molecules since they catalyze the aminoacylation of tRNAs in protein synthesis, an essential process for maintaining cell homeostasis. Nevertheless, ARSs and AIMPs are closely associated with tumor biology [52]. Moreover, according to recent findings in the literature [64], specific ARSs are involved in the induction of unfolded protein response, which has been associated with escape from chemotherapy-induced senescence. This could therefore represent a novel mechanism of resistance to chemotherapy in our models [65]. As a proof of concept, the unfolded protein response process was also captured in the transcriptomic study as one of the most significant hallmarks, and fused data network analysis allowed us to confirm the putative involvement of these processes into a differential oxaliplatin response. In addition, IFNA showed that TCA and OXPHOS genes cluster together with the mitochondrial protein import, a process which was already identified by proteomic approach. Indeed, increased mitochondrial import of proteins involved in ATP synthesis such as ATP5A and ATP5B, together with augmented citrate synthase, a key enzyme of TCA cycle, could mark a shift in tumor metabolism from glycolysis to oxidative phosphorylation pathway (OXPHOS) that could represent a mechanism of oxaliplatin resistance, such as has been unraveled in other tumor models [53]. We could hypothesize that up-regulation of these energy supplier processes guarantees efficient repair of oxaliplatin-induced DNA-damage, since the enzymes involved in this repair consume large amounts of ATP. In fact, targeting ATP synthases has been proposed as a therapeutic strategy to defuel cancer growth. ATP synthase subunit ATP5B has recently been identified as the specific target of apoptolidin A, a glycomacrolide that selectively addressed OXPHOS-dependent cancer [66]. Moreover, data fusion and IFNA evidenced that proteasome proteomic cluster is integrated into a module that contains also genes involved in DNA repair, and highlighted a functional enrichment related to oxidative metabolism and detoxification that connects a previously isolated protein such as PRDX6, a ferroptosis negative regulator [46].

Not all these processes or expression of specific genes were detected by both proteomic and transcriptomic analysis. Two hallmarks, unfolded protein response and PI3K-AKT-mTOR and mTORC1 signaling, were detected by transcriptomic data, but not at the proteomic level. Both were shown to play a significant role in chemotherapy-induced senescence escape [65] and concur with the process highlighted by proteomic results. All this data showed that although transcriptomic and proteomic data were not completely correlated, integrating both analyses could be effective to identify a bona fide mechanism of drug response.

Although CDK4/6 inhibitor palbociclib is not currently used in the clinic to treat CRC, we used it as a proof-of principle of targeted therapy to be assessed in our PDOs. *RAS* wild type RTO7 showed resistance to anti-EGFR drugs, but was extremely sensitive to palbociclib, and differential gene expression analysis showed that it harbors significant MYC activation (GSEA analysis). Analyzing proteomic data, RTO7 has higher levels of Nucleophosmin (NPM), a c-MYC activator [57]. Interestingly, palbociclib has been reported to suppress NPM phosphorylation at threonine 199, thereby reducing cell proliferation in preclinical models of endometrial cancer [67]. *MYC* expression has been shown to correlate with poor prognosis in RAS wild type CRC [68], but probably this is not sufficient to justify response to palbociclib. SWATH studies indicated that this model has a strong activation of several processes involved in proteostasis, among which the whole protein folding TCP-1 ring complex (TRiC) was up-regulated in palbociclib-sensitive PDO. TRiC altered expression was not detected at the mRNA level. However, the unfolded protein response process was one of the most significantly enriched hallmarks found in transcriptomics. Moreover, both omics indicate the same process, while in this case proteomic data also pointed more precisely at the presence of this molecular mechanism. TRiC aids in folding approximately 10% of the proteome, with cytoskeletal proteins actin and tubulin among its best-known substrates, which also include several oncoproteins such as P53 and MYC itself [69]. TRiC proteins were shown to promote cell cycle progression and an invasive phenotype [55] and their overexpression was related with poor prognosis in CRC [70]. Taking all of this into account, altered TRiC complex expression may have prognostic relevance and could be a potential new therapeutic target. In fact, highly proliferative cancer cells increase their dependence on several stress response pathways, including the heat-shock response to alleviate competition among proteins for access to chaperones [71]. These results are reinforced and highlighted after data fusion and IFNA. Indeed, this integrative approach showed that BAG5, detected by RNA-seq, appears to strictly interact with TRiC complex. BAG5 is involved in endoplasmic reticulum stress regulation and unfolded protein response activation [60]. In particular, BAG5 interacts with heat-shock proteins acting as a cochaperone in protein folding [72]. Moreover, some TRiC subunits (TCP1 and CCT5) are among Myc Targets v.1 signature genes (GSEA) and, indeed, TRiC complex overexpression has been correlated with Myc activation [73].

IFNA also highlighted two interesting findings that are in line with a putative Myc addicted phenotype. On one hand, a new module appeared corresponding to cell cycle, mainly represented by RNA-seq data, which was strictly connected to modules involved in proteostasis, mainly represented by proteomic data. On the other hand, the concordance of RNA and protein data indicated an enrichment of metabolic pathways in RTO7, such as pentose phosphate, glycolysis, nucleotides and lipid metabolism, indicating that this PDO is characterized by a high energy demand.

All these results obtained by proteotranscriptomic integration allowed us not only to better identify those functional modules with a direct overlap between omics, but also to define new modules enriched in processes which could have a role in drug response or resistance.

Besides the limitation represented by the number of patients included, which could have underrepresented the heterogeneous mechanisms involved in drug response, we should consider the need to work on functional validation of proteo-transcriptomic data and to validate findings in additional models. In addition, the lack of TME in our models does not allow to study the interplay between cancer cells and their natural microenvironment, thus the possible effect of TME on drug response was not captured in our PDO cultures.

To our knowledge, this is among the few studies employing mass-spectrometry quantitative proteomics by SWATH to characterize PDOs and is the first to integrate proteome study with gene expression profile in the search for potential biomarkers of response/resistance to drug treatment on PDOs. Our findings are confirmed by data fusion and integrative functional network analysis, supporting the inclusion of proteomics in multi-omic characterization and its feasibility in PDOs.

## Conclusions

In conclusion, we have demonstrated the feasibility of building a PDO library from advanced and pretreated CRC patients. We provided a proof-of-concept validation that PDOs, combined with a genomic and proteotranscriptomic approach, can be used to better screen and identify relevant mechanisms involved in sensitivity to anticancer agents in a functional precision medicine context.

## Supplementary Information

**Additional file 1 **.

**Additional file 2** .

**Additional file 3: Supplementary Fig. S1**. PDOs show a heterogeneous growth behavior in culture. Maximum number of days in culture of all models. Each vertical line represents a single passage. Pink: long-term cultures (more than 3 months in culture and more than 10 passages); light pink: do not meet criteria for long-term cultures; blue: short-term cultures (1-3 months in culture and 1-9 passages); light blue: models that do not meet criteria for short-term cultures; ❄ : cryopreservation.

**Additional file 4: Supplementary Fig. S2**. (A) IHC for CDX2 and CK20 antibodies in corresponding tissues. (Scale bar 50 mm, 20X). (B) Lack of expression of MLH1 and PMS2 in tissue corresponding to mCTO50B and RTO2 (Scale bar 50 mm, 20X).

**Additional file 5: Supplementary Fig. S3**. (A) Pearson correlation of SNVs, insertions and deletions across all our cohort. Mutated genes have been filtered per frequency (at least 5% in PDOs). (B) forest plot representing the distribution of r Pearson correlation of mutated genes in each PDO line and corresponding tissue. *p*<0.0001. (C) Linear regression between pathological features and NGS concordance. Tumor cells (%) assessed as the percentage of neoplastic cells with respect to the total amount of viable cells in the tissue sample from which the culture was derived. TSR (tumor-stroma ratio) assessed as the percentage of stroma in a 10x hotspot field, when tumor cells are present in four fields (TSH hotspot) or as average TSR evaluated in up to ten 10X fields. (D) Copy number variation (CNV) profile of ddPCR assays of main driver genes across selected PDOs lines. (Red line: diploid status). CNT33 is a normal genomic DNA sample and is located at the right of the figure in green; samples are depicted in CNV ascending order. The CNV is assumed to follow a Poisson distribution and values represent the estimated number of copies with a 95% confidence interval. Copy number above two means amplification in that region and copy number below two means deletion in that region. (E) Individual plot of first two dimensions using principal componentanalysis of normalized VST showing the distribution of organoid lines and tissues.

**Additional file 6:Supplementary Fig. S4**. (A) Scatterplot for technical replicates of drug screening data. Correlation between the three different technical replicates. Each data point represents the normalized value for an individual organoid line. (B) Log transformed dose-response curves for 5Fluorouracil, oxaliplatin and SN38 and combination respectively and ΔAUC calculation for each line (a negative value or positive indicates presence or absence of additive or synergistic effect, respectively). (C) Log transformed dose-response curves in selected standard drugs. (D) Log transformed dose-response curves in selected non-standard drugs.

**Additional file 7: Supplementary Fig. S5**. (A) Log2 TPM+1 of CDK4 and CDK6 expression in RTO7, BRD2 and BRD4 expression in RTO2, c-MYC and SMAD4 in RTO7 compared with the other cultures. (B) PTEN IHC in MSI PDOs and RTO7 as positive control. (C) Principal component analysis of protein expression showing the distribution of organoid lines and tissues. Variance absorption from PC3 and PC4: 21.98%. (D) Log2 protein abundance (PA) of TriC complex proteins in RTO7 versus RTO2/mCTO66S3. (E) Western blot analysis of TCP1, CCT2 and CCT8 proteins. GADPH is included as control. TPM: transcripts per million; PA: protein abundance.

**Additional file 8: Supplementary Fig. S6**. (A) Correlation between gene (Log2 TPM) and protein (Log2 PA) expression. Differentially expressed proteins have been matched with corresponding genes. (B) Log2 TPM+1 of TRiC complex and ARSs gene expression in palbociclib (upper panel) andoxaliplatin (lower panel) comparisons. TPM: transcripts per million; PA: protein abundance.

**Additional file 9: Supplementary Fig. S7**. Differentially expressed proteins mapped in KEGG metabolic pathways (hsa01100) in the palbociclib comparison related to the metabolic enrichment group. In red those pathway reactions catalyzed by proteins identified by RNA-seq only, in blue those by proteomics only, in green those identified by both omics.

**Additional file 10: Supplementary Table S1.** All sample characteristics are represented among our PDOs cohort. *P* value refers to two-sided Fisher’s exact test for pre-treated and RAS/RAF status variables and to Chi-Square test for PTL and site of biopsy, being Chi-square results 0,4759, 2 and 2,671, 2 respectively.

## Data Availability

We are in process of submitting all genomics, transcriptomics, and proteomics raw data in public repositories.

## Abbreviations

AF: Allelic fraction
ARSs: Aminoacyl-tRNA synthetase
AUC: Area under the curve
CRC: Colorectal cancer
CS: Citrate synthase
ddPCR: Droplet-digital PCR
EMT: Epithelial-mesenchymal transition
FDR: False discovery rates
FFPE: Formalin-fixed paraffin embedded
GSEA: Gene set enrichment analysis
H&E: Hematoxylin and eosin
IHC: Immunohistochemistry
IFNA: Integrative functional network analysis
LOH: Loss of heterozygosity
MSC: Multi-tRNA synthetase complex
MSI: Microsatellite instability
NGS: Next-generation sequencing
NPM1: Nucleophosmin
OXPHOS: Oxidative phosphorylation
PCA: Principal component analysis
PDOs: Patient-derived organoids
SNVs: Single nucleotide variants
SWATH-MS: Sequential window acquisition of all theoretical mass spectrometry
TCA: Tricarboxylic acid cycle
TME: Tumor microenvironment
TRiC: T-complex protein Ring Complex
VAFs: Variant allele frequencies
