# Supplementary figures and images for: “Proteotranscriptomic analysis of advanced colorectal cancer patient derived organoids for drug sensitivity prediction”
Source: J Exp Clin Cancer Res. 2023 Jan 6;42:8. doi: 10.1186/s13046-022-02591-z (PMC9817273; doi:10.1186/s13046-022-02591-z)

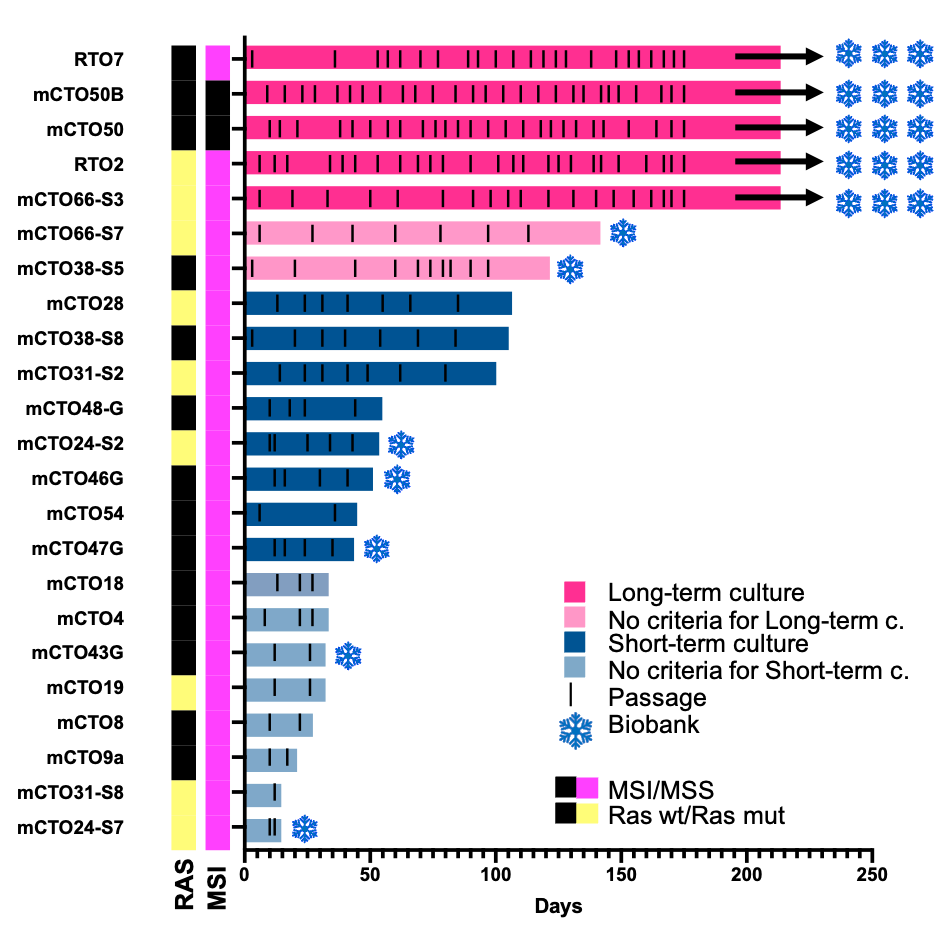

Supplement: Supplementary file 3 — Additional file 3: Supplementary Fig. S1. PDOs show a heterogeneous growth behavior in culture. Maximum number of days in culture of all models. Each vertical line represents a single passage. Pink: long-term cultures (more than 3 months in culture and more than 10 passages); light pink: do not meet criteria for long-term cultures; blue: short-term cultures (1-3 months in culture and 1-9 passages); light blue: models that do not meet criteria for short-term cultures; ❄ : cryopreservation. [file 13046_2022_2591_MOESM3_ESM.png]

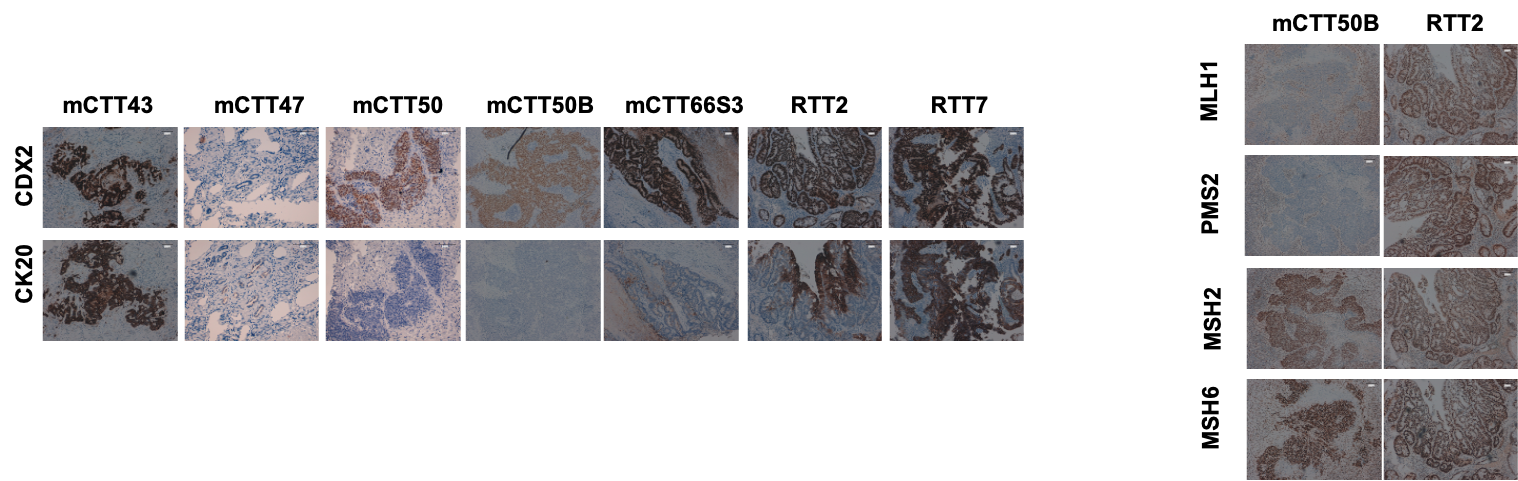

Supplement: Supplementary file 4 — Additional file 4: Supplementary Fig. S2. (A) IHC for CDX2 and CK20 antibodies in corresponding tissues. (Scale bar 50 mm, 20X). (B) Lack of expression of MLH1 and PMS2 in tissue corresponding to mCTO50B and RTO2 (Scale bar 50 mm, 20X). [file 13046_2022_2591_MOESM4_ESM.png]

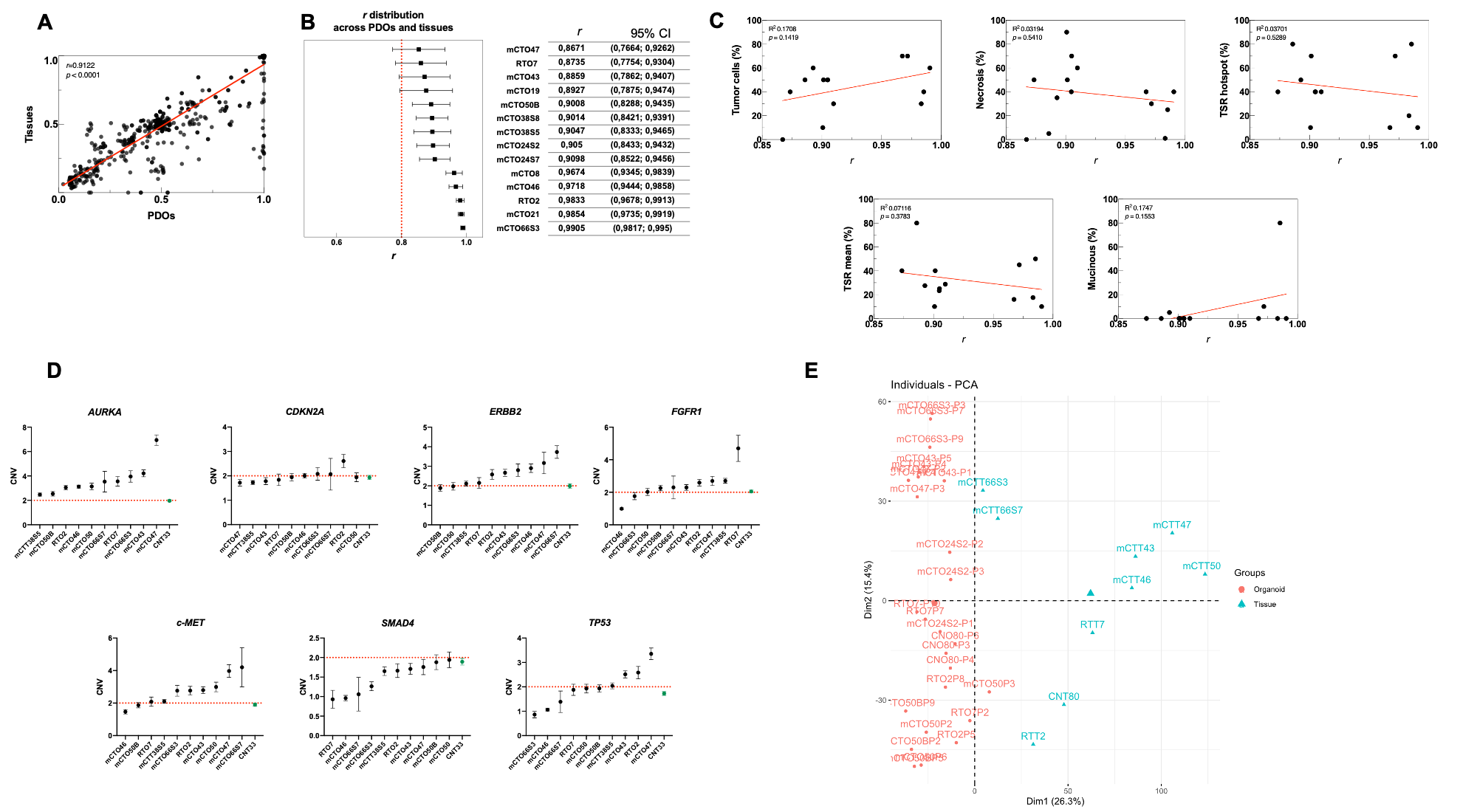

Supplement: Supplementary file 5 — Additional file 5: Supplementary Fig. S3. (A) Pearson correlation of SNVs, insertions and deletions across all our cohort. Mutated genes have been filtered per frequency (at least 5% in PDOs). (B) forest plot representing the distribution of r Pearson correlation of mutated genes in each PDO line and corresponding tissue. p<0.0001. (C) Linear regression between pathological features and NGS concordance. Tumor cells (%) assessed as the percentage of neoplastic cells with respect to the total amount of viable cells in the tissue sample from which the culture was derived. TSR (tumor-stroma ratio) assessed as the percentage of stroma in a 10x hotspot field, when tumor cells are present in four fields (TSH hotspot) or as average TSR evaluated in up to ten 10X fields. (D) Copy number variation (CNV) profile of ddPCR assays of main driver genes across selected PDOs lines. (Red line: diploid status). CNT33 is a normal genomic DNA sample and is located at the right of the figure in green; samples are depicted in CNV ascending order. The CNV is assumed to follow a Poisson distribution and values represent the estimated number of copies with a 95% confidence interval. Copy number above two means amplification in that region and copy number below two means deletion in that region. (E) Individual plot of first two dimensions using principal componentanalysis of normalized VST showing the distribution of organoid lines and tissues. [file 13046_2022_2591_MOESM5_ESM.png]

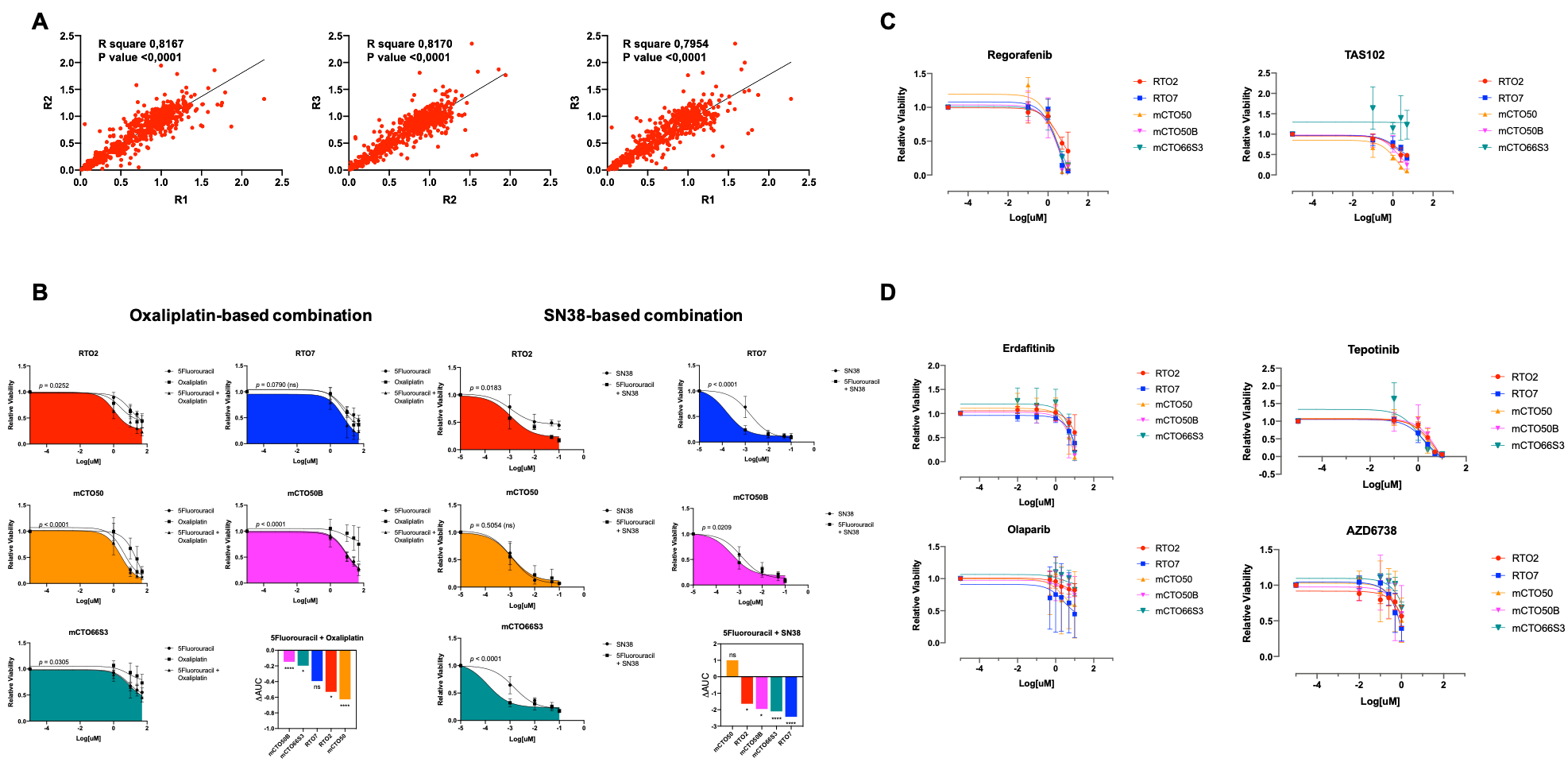

Supplement: Supplementary file 6 — Additional file 6:Supplementary Fig. S4. (A) Scatterplot for technical replicates of drug screening data. Correlation between the three different technical replicates. Each data point represents the normalized value for an individual organoid line. (B) Log transformed dose-response curves for 5Fluorouracil, oxaliplatin and SN38 and combination respectively and ΔAUC calculation for each line (a negative value or positive indicates presence or absence of additive or synergistic effect, respectively). (C) Log transformed dose-response curves in selected standard drugs. (D) Log transformed dose-response curves in selected non-standard drugs. [file 13046_2022_2591_MOESM6_ESM.png]

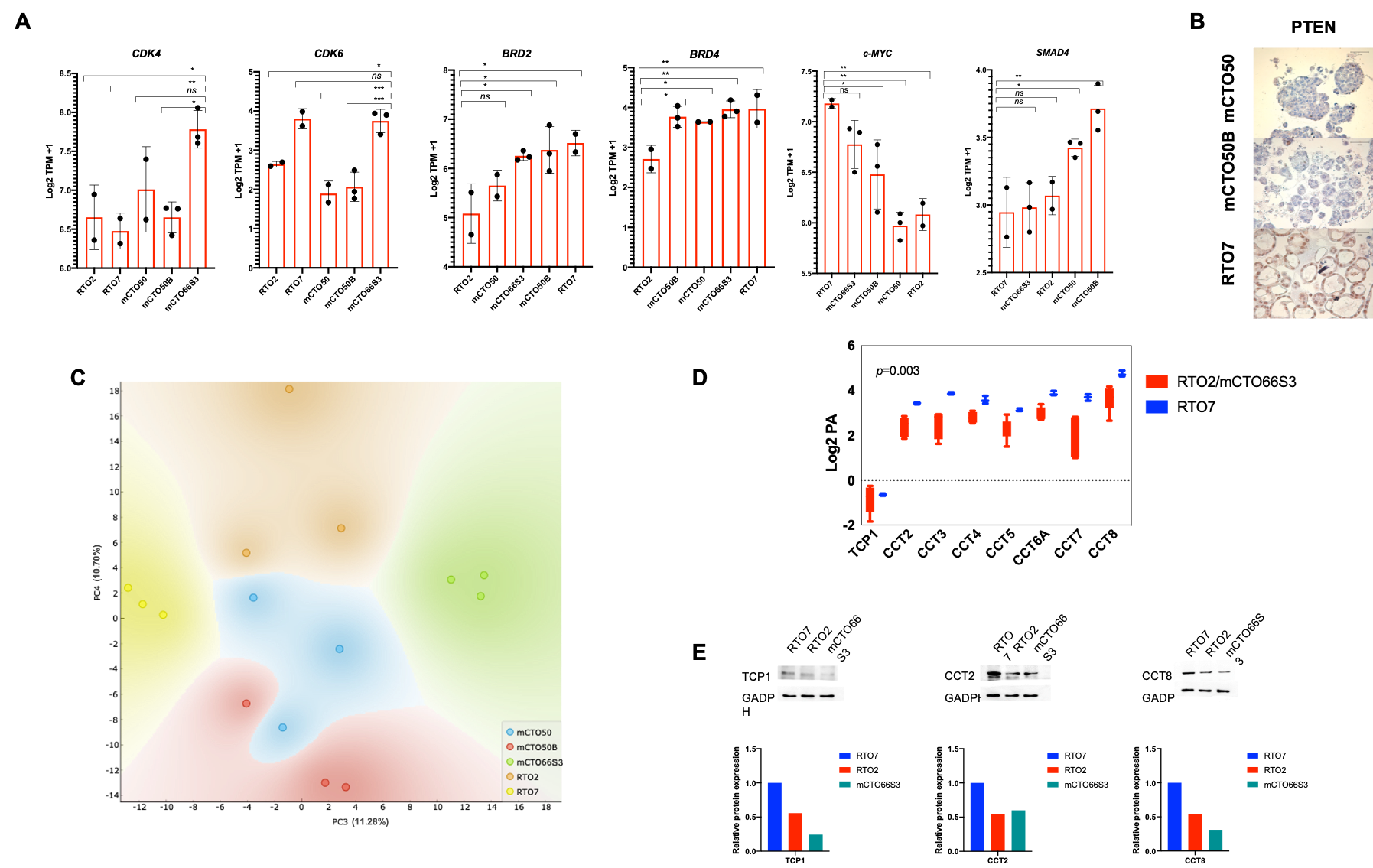

Supplement: Supplementary file 7 — Additional file 7: Supplementary Fig. S5. (A) Log2 TPM+1 of CDK4 and CDK6 expression in RTO7, BRD2 and BRD4 expression in RTO2, c-MYC and SMAD4 in RTO7 compared with the other cultures. (B) PTEN IHC in MSI PDOs and RTO7 as positive control. (C) Principal component analysis of protein expression showing the distribution of organoid lines and tissues. Variance absorption from PC3 and PC4: 21.98%. (D) Log2 protein abundance (PA) of TriC complex proteins in RTO7 versus RTO2/mCTO66S3. (E) Western blot analysis of TCP1, CCT2 and CCT8 proteins. GADPH is included as control. TPM: transcripts per million; PA: protein abundance. [file 13046_2022_2591_MOESM7_ESM.png]

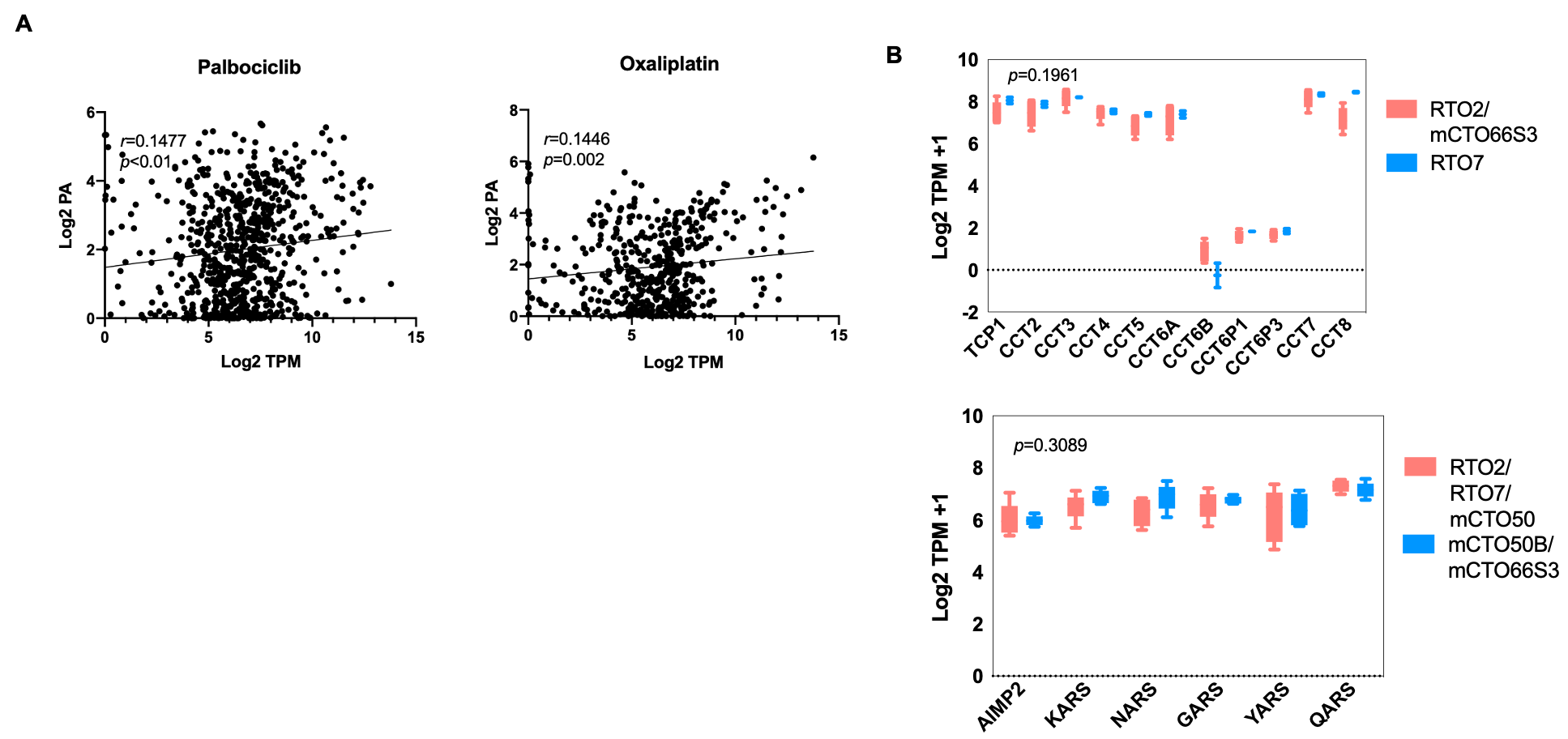

Supplement: Supplementary file 8 — Additional file 8: Supplementary Fig. S6. (A) Correlation between gene (Log2 TPM) and protein (Log2 PA) expression. Differentially expressed proteins have been matched with corresponding genes. (B) Log2 TPM+1 of TRiC complex and ARSs gene expression in palbociclib (upper panel) andoxaliplatin (lower panel) comparisons. TPM: transcripts per million; PA: protein abundance. [file 13046_2022_2591_MOESM8_ESM.png]

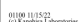

Supplement: Supplementary file 9 — Additional file 9: Supplementary Fig. S7. Differentially expressed proteins mapped in KEGG metabolic pathways (hsa01100) in the palbociclib comparison related to the metabolic enrichment group. In red those pathway reactions catalyzed by proteins identified by RNA-seq only, in blue those by proteomics only, in green those identified by both omics. [file 13046_2022_2591_MOESM9_ESM.png]
