# Supplementary material for: “Proteotranscriptomic analysis of advanced colorectal cancer patient derived organoids for drug sensitivity prediction”
Source: J Exp Clin Cancer Res. 2023 Jan 6;42:8. doi: 10.1186/s13046-022-02591-z (PMC9817273; doi:10.1186/s13046-022-02591-z)
Supplement: Supplementary file 10 — Additional file 10: Supplementary Table S1. All sample characteristics are represented among our PDOs cohort. P value refers to two-sided Fisher’s exact test for pre-treated and RAS/RAF status variables and to Chi-Square test for PTL and site of biopsy, being Chi-square results 0,4759, 2 and 2,671, 2 respectively. [file 13046_2022_2591_MOESM10_ESM.docx]

Supplementary Table S1

All sample characteristics are represented among our PDOs cohort. *P* value refers to two-sided Fisher’s exact test for pre-treated and RAS/RAF status variables and to Chi-Square test for PTL and site of biopsy, being Chi-square results 0,4759, 2 and 2,671, 2 respectively.

|  | PDOs n(%)/patients n(%) | *p* |
| --- | --- | --- |
| PDOs Lines (n) | 29/22 |  |
| Longitudinal samples | 5/2 |  |
| Multi-samples | 14/7 |  |
| Pre-treatment biopsies (1 UKn)*  Post-treatment* biopsies | 10(34)/9(40)  18(66)/13(60) | 0.1895 |
| 1^st^ line (1 n/a, 1 UKn)  Before  After | 21(72)/16(72)  7(33)/6(37)  14(67)/11(63) | 0.2630 |
| 2^nd^ line  Before  After | 3(10)/3(13)  2(66)/2(66)  1(34)/1(34) |  |
| ≥ 3^rd^ line (after) | 3(10)/1(4) |  |
| PTL (1 UKn*): right  PTL: left  PTL: rectum | 6(20)/5(23)  12(41)/8(36)  10(39)/8(36) | 0.7883 |
| Site of biopsy: Primary  Liver mets  Non liver mets (node, brain, adrenal) | 2(7)/2(9)  25(86)/18(82)  2(7)/2(9) | 0.4837 |
| RAS/RAF wt (2 UKn)  RAS/RAF mut | 13(45)/11(50)  16(55)/10(45) | >0.9999 |

UKn: Unknown (missed clinical information)

n/a: not applicable (refers to locally advanced patient)

*One patient was biopsied before and after treatment, at disease progression.
